# MAJEC: unified gene, isoform, and locus-level transposable element quantification from RNA-seq

**DOI:** 10.1093/bioinformatics/btag737

**Published:** 2026-10-05

**Authors:** Tian-Yeh Lim, Ari J Firestone

**Affiliations:** Calico Life Sciences LLC, South San Francisco, CA 94080, United States; Calico Life Sciences LLC, South San Francisco, CA 94080, United States

## Abstract

**Motivation:**

The study of transposable elements (TEs) has become increasingly central to fields such as cancer biology, immunology, and aging. Accurately quantifying disease- or laboratory-mediated perturbations in these elements is critical to support this expanding research, yet current RNA-seq pipelines struggle with the pervasive overlap between TEs and protein-coding genes. Existing tools either aggregate to the subfamily level with no locus resolution (TEtranscripts), or provide locus-level quantification without modeling gene overlap (Telescope), with the latter attributing over 40% of TE signal to the 1.1% of loci that overlap gene exons.

**Results:**

We present Momentum Accelerated Junction Enhanced Counting (MAJEC), a unified Expectation-Maximization (EM) framework that jointly quantifies genes, transcript isoforms, and individual TE loci from BAM alignments in a single pass. Splice junction evidence informs transcript-level priors, enabling MAJEC to probabilistically distinguish genic from TE-derived reads. This approach was independently validated against Salmon and RSEM on isoform quantification benchmarks. The joint feature space reduces exon-overlap contamination of locus-level TE estimates from 43% of total signal (Telescope) to 5% (MAJEC), while preserving subfamily-level accuracy (differential expression *r* = 0.987 versus TEtranscripts). Using paired biological vignettes, we demonstrate that MAJEC correctly resolves both the false TE reactivation artifacts endemic to TE-only models, and the false gene upregulation artifacts that occur when heuristic rules misassign genuine intragenic TE transcription. MAJEC simultaneously produces the isoform and locus-level resolution that TEtranscripts lacks, with greater accuracy than Telescope, and runs faster than either.

**Availability and implementation:**

MAJEC is available at https://github.com/calico/majec and via PyPI/Bioconda; analysis code and data are deposited at https://doi.org/10.5281/zenodo.19224157.

## 1 Introduction

RNA-seq quantification for genes and their isoforms versus transposable elements (TEs) has bifurcated into two largely independent ecosystems. On the gene side, several distinct method classes coexist: counting-based tools such as featureCounts (Liao *et al*. 2014) and HTSeq (Anders *et al*. 2015) tally reads for unambiguous gene-level quantification; alignment-based expectation-maximization frameworks such as RSEM (Li *et al*. 2010, Li and Dewey 2011) resolve multi-mapping reads at the isoform level from aligned reads; and alignment-free tools such as Salmon (Patro *et al*. 2017) and Kallisto (Bray *et al.* 2016) estimate isoform abundance through lightweight sequence-level modeling. For TEs, a separate set of specialized tools has emerged to address the multi-mapping problem inherent in quantifying repetitive sequences: TEtranscripts (Jin *et al*. 2015) distributes multi-mapped reads across TE subfamilies using an expectation-maximization (EM) algorithm, Telescope (Bendall *et al*. 2019) extends this to locus-level resolution with a Bayesian reassignment model and SQuIRE (Yang *et al*. 2019) adds locus-level TE quantification alongside a separate gene-expression estimate. In practice, obtaining accurate quantification across transcript isoforms, genes, and individual TE loci still requires combining tools that each address only part of the problem, with different alignment requirements, annotation formats, and statistical models; and none resolve gene-TE read overlap within a single model.

Approximately 45% of the human genome derives from TEs (Lander *et al*. 2001), and interest in TE biology has intensified as roles for TE reactivation have emerged in cancer, aging, innate immunity, and epigenetic therapy (Chiappinelli *et al*. 2015, Roulois *et al*. 2015, Burns 2017, De Cecco *et al*. 2019, Griffin *et al*. 2021). A growing number of studies require not just subfamily-level differential expression—“are L1PA7 elements upregulated as a class?”—but locus-level resolution: which *specific* TE insertions are reactivated, where are they in the genome, and what is their relationship to overlapping genes? This granularity is essential for applications such as identifying TE-derived biomarkers of epigenetic drug response, characterizing chimeric TE-gene transcripts, and understanding the regulatory consequences of individual TE insertions.

TEtranscripts, the most widely used TE quantification tool, provides robust subfamily-level differential expression analysis but does not resolve individual TE loci. It also carries a substantial computational cost, which becomes a practical bottleneck for large experiments. Telescope addresses the locus resolution gap but introduces a different limitation: it operates entirely within a TE-only feature space with no access to gene annotations. Since TE insertions are densely embedded within gene bodies—in introns, untranslated regions, and occasionally exons—Telescope has no mechanism to distinguish reads originating from an independently transcribed TE from reads belonging to the host gene’s transcript that happen to overlap a TE annotation. We show that this is not a minor edge case: nearly half of Telescope’s total TE signal originates from exon-overlapping loci, which constitute only 1.1% of all TE features. Thus, a 40-fold enrichment for locus-level TE expression is potentially driven by genic signal at exon-TE boundaries.

TEtranscripts mitigates this problem at the subfamily level through a hard-coded rule that assigns uniquely mapped reads overlapping both a gene exon and a TE annotation exclusively to the gene. This conservative heuristic is effective—Momentum Accelerated Junction Enhanced Counting (MAJEC) and TEtranscripts agree at *r* = 0.995 at the subfamily level despite completely different algorithmic approaches. However, the rule does not generalize to locus-level quantification, where the question is not whether a read belongs to “genes” or “TEs” in aggregate but whether a specific read at a specific locus reflects gene or TE transcription. At this resolution, a rigid rule that unconditionally privileges one feature type over the other cannot be correct in both directions: it will suppress genuine TE signal at genic loci just as readily as it suppresses false TE signal.

A joint model that quantifies genes and TEs simultaneously in a shared feature space would resolve this problem by allowing reads to be assigned to whichever feature—gene or TE—is best supported by the data at each locus. Such a model requires accurate isoform-level quantification of the genic component, since reads must be assigned not just to genes but to specific transcripts in order to determine whether they are consistent with the gene’s splice structure or are better explained by independent TE transcription. Splice junction-spanning reads provide particularly strong evidence: a read spanning a known splice junction of a transcript can be confidently assigned to that transcript rather than to an overlapping TE. Junction evidence thus serves a dual purpose—it improves isoform quantification accuracy, and it provides the mechanistic basis for distinguishing genic from TE-derived signal at overlapping loci.

Here we present MAJEC, a unified framework that jointly quantifies genes, transcript isoforms, and individual TE loci from aligned RNA-seq reads (BAM files) in a single analysis. MAJEC operates a locus-level EM algorithm—preserving the individual TE resolution of Telescope—within a joint gene + TE feature space where all annotated transcripts and all annotated TE loci compete for reads simultaneously. Junction-spanning reads inform transcript-level priors through a completeness penalty system that downweights transcripts lacking expected splice junctions, improving isoform accuracy on complex genes and providing high-confidence evidence for genic read assignment at gene-TE overlaps.

We validate MAJEC’s isoform quantification against Salmon on synthetic transcriptomes [Sequins (Hardwick *et al*. 2016, Dong *et al*. 2023)] and against both Salmon and RSEM on complex real transcriptomes [LongBench (You *et al*. 2025)], demonstrating that junction-informed priors improve accuracy specifically where isoform structure is ambiguous. We show that MAJEC matches TEtranscripts at the subfamily level for differential expression (*r* = 0.987) while providing locus-level resolution that TEtranscripts cannot, and we demonstrate through paired vignettes that the joint model correctly resolves cases where existing tools fail in opposite directions—Telescope falsely reporting TE reactivation from genic reads, and TEtranscripts falsely inflating gene expression from TE-derived reads. MAJEC completes these analyses faster than the specialized TE tools it replaces, from a BAM alignment that most RNA-seq workflows already produce.

## 2 Materials and methods

### 2.1 Overview

MAJEC quantifies genes, transcript isoforms, and individual TE loci from coordinate-sorted BAM files in a single analysis. The pipeline proceeds in five stages: (i) initial read assignment of uniquely mapping reads via featureCounts, with fractional apportionment when reads overlap multiple annotated features, (ii) extraction and scoring of splice junction evidence, (iii) featureCounts-based generation of multi-mapper equivalence classes, (iv) a two-phase expectation-maximization (EM) algorithm operating on a joint gene + TE feature space, and (v) confidence scoring and output generation.

MAJEC requires as input a coordinate-sorted BAM file from any splice-aware aligner [e.g. STAR (Dobin *et al*. 2013) or HISAT2], a gene annotation GTF (Ensembl or GENCODE), and a TE annotation file [RepeatMasker (Smit *et al*. 2013)]. For TE quantification, the upstream aligner must retain multi-mapping reads (e.g. STAR with --outFilterMultimapNmax 100 or higher); this is the same requirement as TEtranscripts and Telescope. MAJEC runs on standard Linux workstations with 32–64 GB RAM and does not require HPC infrastructure.

### 2.2 Feature annotation and joint model design

MAJEC constructs a unified feature space containing all annotated transcript isoforms from the gene GTF and all annotated TE loci from RepeatMasker. In this joint model, a read overlapping both a gene exon and a TE annotation competes between the two feature types—the EM assigns it to whichever feature is best supported by the available evidence. This is the key architectural difference from existing TE tools.

The EM operates at individual transcript and TE locus resolution—preserving the locus-level granularity. Subfamily-level and gene-level counts are derived by *post hoc* aggregation of locus-level estimates.

Subset and superset relationships between transcripts are identified during annotation preprocessing. A transcript whose set of annotated splice junctions is entirely contained within another transcript’s junction set is flagged as a potential subset isoform, subject to the subset penalty system described below.

To accurately apply these penalties, MAJEC employs an annotation preprocessing step. The pipeline constructs a deterministic index of all expected splice junctions, categorizing each as either unique to a specific isoform or as a site of direct splice competition (e.g. shared donor but alternative acceptor sites). Furthermore, rather than uniformly penalizing subset transcripts, MAJEC utilizes interval-tree logic to identify “unambiguous exonic territories”—genomic coordinates strictly unique to the subset isoform. By categorizing the structural basis of these unique regions (such as retained introns or alternative terminal extremities), the pipeline extracts isolated regions to serve as “rescue features.” During quantification, coverage across these precise unique intervals can empirically validate a subset’s expression, rescuing it from being erroneously zeroed out by its superset.

### 2.3 Initial read assignment

Equivalence classes are built from two sequential featureCounts runs. Initial counts for reads with no mapping ambiguity are collected first. After filtering the BAM for multi-mapped reads, a second set of equivalence classes is generated with fractional counting enabled for reads mapping to multiple features. MAJEC modifies the default featureCounts fractional counting behavior by pooling all features across all alignment positions for each multi-mapped read, distributing the count equally across the union of overlapping features rather than independently at each alignment position. This ensures that the initial count for a multi-mapper reflects the full set of candidate features before EM resolution. Unlike the default multi-overlap mode in featureCounts, MAJEC’s union-based fractional assignment strictly conserves total read mass: each read contributes exactly one count distributed across all candidate features. For paired-end data, counting is performed at the fragment level, and strandedness is handled according to the library preparation protocol. To ensure memory efficiency, the featureCounts processes are streamed via FIFO pipes, allowing for the concurrent processing of multiple BAM files.

### 2.4 Junction evidence and prior adjustments

Before the EM begins, initial counts from featureCounts are modulated by a series of evidence-based priors computed from splice junction observations. Junction-spanning reads are extracted from the BAM file and mapped to annotated splice sites in the GTF, providing per-sample evidence for which transcripts are actively spliced.

Four prior adjustments are applied sequentially.

#### 2.4.1 Junction evidence boost

Transcripts receive additional counts proportional to their splice junction support, weighted by junction uniqueness. For a junction *j* shared by *n* transcripts, the evidence contributed to each transcript is scaled by (1/*n*)^*d*^, where *d* is a decay exponent (default 1.0). This ensures that junctions unique to a single transcript provide strong evidence, while shared junctions contribute proportionally less. The magnitude of the boost is controlled by a global junction weight parameter (default 3.0).

#### 2.4.2 Transcript support level penalty

Transcripts are optionally down-weighted based on their GENCODE/Ensembl Transcript Support Level (TSL) annotation, ranging from [default = 1.0 (TSL1, highest confidence) to 0.3 (TSL5, lowest confidence), with 0.8 for transcripts with no TSL annotation]. Use and weighting of TSL-based penalties are user configurable.

#### 2.4.3 Junction completeness penalty

For each multi-exon transcript, MAJEC compares observed junction-spanning read counts to the expected coverage based on the transcript’s median junction depth. Junctions with substantially lower coverage than expected receive a *Z*-score-based penalty, incorporating an overdispersion parameter (default 0.05) that models biological and technical variance. Terminal junctions (5′ or 3′ ends) receive relaxed penalties when appropriate for the library type (e.g. 3′ dropout in poly-dT libraries). The completeness penalty ranges from 1.0 (all expected junctions observed at expected coverage) to a configurable minimum (default 0.0001). This penalty is the primary driver of MAJEC’s isoform accuracy advantage, as demonstrated by feature ablation (see Section 3).

#### 2.4.4 Subset isoform penalty

Transcripts identified as potential subsets of longer isoforms during annotation preprocessing are evaluated in two stages. First, an annotation-based penalty compares the subset’s shared junction coverage to the expected contribution from its superset transcripts; if the observed coverage does not exceed the expected superset contribution (controlled by a threshold parameter, default 1.25), an initial penalty is applied. Second, when unique exonic territory data is available, direct read coverage in the subset’s unique regions is compared to coverage in shared regions, providing data-driven validation or override of the annotation-based penalty. The subset penalty floor (default 0.001) controls the extent of subset transcripts suppression when the evidence is ambiguous.

The adjusted counts after all four prior stages serve as the initial estimates for the EM algorithm.

### 2.5 Two-phase EM algorithm

MAJEC employs a hierarchical two-phase EM that processes unique and multi-mapping reads separately, recognizing that they represent fundamentally different levels of uncertainty.

In Phase 1, uniquely mapped reads are assigned to transcripts and TE loci based on the prior-adjusted initial estimates. Because these reads have unambiguous genomic locations, the only ambiguity is among overlapping features at the same locus (e.g. overlapping isoforms, or a gene exon overlapping a TE annotation). In Phase 2, multi-mapping reads are iteratively allocated using a combination of the fixed unique-mapper estimates from Phase 1 and the updated multi-mapper probabilities from the preceding EM iteration.

The E-step allocates reads within each equivalence class (a group of reads sharing the same set of possible feature assignments) proportionally to current expression estimates:

For transcript *t* in equivalence class *C*:


$$\theta_{t,i+1}={\text{reads}}_{C}\times \frac{\theta_{t,i}}{\sum_{j\in C}\theta_{j,i}}$$

The combined estimate after both phases is $\theta_{\text{total},t,i+1}=\theta_{\text{unique},t,i+1}+\theta_{\text{multi},t,i+1}$.

#### 2.5.1 Momentum acceleration

MAJEC accelerates convergence using expression-level grouped momentum. After an initial burn-in period, velocity vectors (*θ*[*t*, *i*] − *θ*[*t*, *i *− 1]) are computed per transcript and scaled by expression-group-specific momentum factors (default: 1.5 for low-expression, 1.0 for medium, 0.7 for high-expression transcripts). Stability safeguards include oscillation detection, maximum step-size limits, and automatic fallback to standard EM upon detection of instability.

#### 2.5.2 Convergence

The EM iterates until relative change, absolute change, and total count stability all fall below adaptive thresholds, or until the iteration cap is reached (default 100). In practice, convergence occurred within 15–17 iterations across all tested samples; the iteration cap is a safety net rather than a tuning parameter.

#### 2.5.3 Effective length correction

Transcript effective lengths are computed from the empirical fragment length distribution (estimated from the BAM file or provided by the user) using a Normal distribution model of mappable positions along each transcript.

### 2.6 Confidence metrics

MAJEC reports several per-transcript and per-group confidence metrics alongside the count estimates.

#### 2.6.1 Distinguishability score

A composite metric combining the fraction of reads with unambiguous assignment (unique mappers plus unique junction evidence from multi-mappers) with a measure of how well the transcript was separated from competitors within the ambiguous read fraction (ambiguous_distinguishability):


$${\text{distinguishability}}_{t}={\text{certain}\_\text{fraction}}_{t}\times 1.0+{\text{uncertain}\_\text{fraction}}_{t}\times {\text{ambiguous}\_\text{distinguishability}}_{t}$$

Scores range from 1.0 (perfectly distinguishable) to 0.0 (highly ambiguous).

#### 2.6.2 Assignment entropy

Shannon entropy of the read source distribution for each transcript, converted to a confidence score: confidence[*t*] = 1/(1 +entropy[*t*]).

#### 2.6.3 Discord score

A measure of EM revision magnitude, computed as an expression-normalized log reduction from pre-EM to post-EM counts. For each transcript with pre-EM count *p* > post-EM count *q*:


$$d=\log_{10}\left(\frac{p}{q+1}\right)$$

This raw discord is normalized by an expression-dependent scale factor

$s=1+\frac{\log_{10}\left(p+10\right)}{4}$ , ensuring that proportionally equivalent revisions receive similar scores regardless of expression level. A saturating transform bounds the final score on [0, 4):


$$\text{discord}=\frac{4(d/s)}{1+(d/s)}$$

Transcripts where the EM did not reduce counts receive a score of zero. High discord combined with unique junction support identifies a small population (∼2% of high-discord transcripts) representing potential EM over-suppression candidates.

#### 2.6.4 Group-level metrics

For genes and TE subfamilies, expression-weighted averages of transcript-level scores are computed, along with an inter-group competition metric that distinguishes ambiguity from complex internal splicing (low inter-group competition) versus ambiguity from external paralogs or gene-TE overlap (high inter-group competition).

### 2.7 Output

MAJEC produces transcript-level and TE locus-level count estimates, which are aggregated to gene-level and TE subfamily-level counts by summation. MAJEC outputs transcript-level and TE locus-level count estimates as tab-delimited files. Optional companion utilities ingest these results into a SQLite database for downstream querying, extract DESeq2-compatible count matrices at the gene, isoform, and TE locus levels, and generate interactive HTML reports (Plotly-based) for isoform-level visualization of junction evidence, expression proportions, and confidence metrics.

### 2.8 Benchmarking datasets

The **Sequins v2.4 spike-in dataset** comprises 160 synthetic RNA isoforms across 76 genes with known ground truth concentrations in two mixes (Mix A and Mix B), spiked into human RNA and sequenced as paired-end short reads. Reads pairs (38.9–61.6 M) were aligned with STAR 2.7.11b to a hybrid hg38 + Sequin chromosome genome using --outFilterMultimapNmax 100 and gene annotations from refGene (appropriate for the synthetic transcript models). MAJEC, Salmon 1.10.3 (with --gcBias --seqBias), RSEM 1.2.28 (using the same hybrid genome and reference [rsem-prepare-reference --gtf --star --star-sjdbOverhang 80]) featureCounts 2.1.1 (raw fractional counts, pre-EM), and long-read quantification were compared. Accuracy was assessed using median absolute log2 fold error (with pseudocount of 1) and Pearson correlation in log-transformed space.

The **LongBench dataset** is composed of Eight ENCODE lung cancer cell lines (SHP-77, NCI-H146, NCI-H526, NCI-H2228, NCI-H69, HCC827, NCI-H211, NCI-H1975) with matched short-read (55–71 M read-pairs) and long-read data were used for isoform quantification benchmarking. Short reads were aligned with STAR 2.7.11b using --outFilterMultimapNmax 100 against the GENCODE v44 annotation (Frankish *et al*. 2021). Long-read data from three platforms—ONT direct RNA (dRNA), PacBio Iso-Seq, and ONT cDNA—were aligned with pbmm2 1.9.0 (--preset ISOSEQ) and quantified with IsoQuant 3.9.0 to serve as approximate ground truth, along with their consensus average (mean across platforms). MAJEC, Salmon 1.10.3 (with --gcBias --seqBias), and RSEM 1.2.28 were compared.

Quantification error of each tool’s TPM measurements normalized to 10 M was computed as |log1p(observed) - log1p(truth)|, where truth was long-read data normalized to counts-per-10-million. For long-read data count and transcript space are considered equivalent. A transcript–cell line pair entered the analysis if the long-read consensus reached 1, or the highest of the three short-read estimates (MAJEC, Salmon, RSEM) reached 1, per 10 M. The primary comparison metric was the superiority rate: the fraction of transcripts where MAJEC had lower error than the comparator, with ties split equally. Statistical significance was assessed by Wilcoxon signed-rank test on paired error differences. Analyses were stratified by MAJEC’s junction penalty classification (none, incomplete, subset, or both penalties below 0.2), by ground truth expression level, and by transcript detection (sensitivity, precision, F1).

Intrinsic transcript properties analysis was conducted on per-transcript accuracy outcomes using the full LongBench benchmark modeled against intrinsic transcript features. The analysis was restricted to protein-coding and lncRNA transcripts using the GENCODE v44 biotypes. For each transcript in each cell line, a “win” was using the superiority metric and error calculated as above against the consensus long-read ground truth (mean of PacBio, ONT, and dRNA). Intrinsic features (isoforms per gene, exon count, spliced transcript length, and GC content) were computed from the GENCODE v44 annotation and the matching human genome, with log10 expression (long-read consensus) as a covariate. Feature effects were estimated by multivariable logistic regression on decided cases (exact ties excluded) with all predictors standardized (variance inflation factors < 1.8), and by ordinary least squares on the continuous error difference; standard errors were made robust by clustering on gene (32 403 clusters) to account for transcripts observed across the eight cell lines.

For sequencing depth analysis, 3 lines (SHP-77, NCI-H2228, NCI-H146) were downsampled to 7.5, 15, 30, and 55 M read pairs with seqtk 1.5-r133 (Li 2025; fixed seed, applied identically to both mates). Each subset was aligned with STAR and quantified with both MAJEC and Salmon as above from the identical read subset. Isoforms were classified by their long-read intra-gene structure as dominant, minor, or rare (rare = a minor isoform contributing <5% of its gene’s long-read signal), and median log-fold error and detection sensitivity using a threshold of 1 in the per-10-million—normalized count were computed on the long-read—confirmed isoforms within each stratum.

An independent replication was performed on a T-cell dataset GSE229972 (Woolley *et al*. 2025) with matched short-read (58.3–69.4 M read-pairs) and PacBio Iso-Seq data from three timepoints. Short reads were aligned with STAR 2.7.11b against GENCODE v29 annotations; PacBio reads were aligned with pbmm2 1.9.0 (PacificBiosciences, n.d.), a PacBio-native frontend for the minimap2 (Li 2018) and quantified with IsoQuant 3.9.0. (Prjibelski *et al*. 2023).

#### 2.8.1 TE benchmarking (decitabine treatment)

Stranded paired-end RNA-seq (28.0–35.5 M read-pairs per sample) was generated from FaDu (ATCC, HTB-43) head and neck squamous carcinoma cells treated with DMSO or 1 µM decitabine for 7-days in triplicate. Reads were aligned with STAR 2.6.1a with --outFilterMultimapNmax 100 --winAnchorMultimapNmax 200, retaining the alignment used for the original TEtranscripts analysis. Gene annotations were from refGene (hg38refGene); TE annotations were from RepeatMasker (hg38_rmsk_TE, locus-level). MAJEC was run in two modes: joint (gene + TE feature space) and TE-only (identical EM without gene features). Results were compared to SQuIRE 0.9.9.92, TEtranscripts 2.2.3, Telescope 1.0.3 (--reassign_mode conf), and an additional development branch of Telescope with a stranded mode (1.0.3 + 48.g4cf1859) run with the same command plus --stranded_mode RF. MAJEC, TEtranscripts, and both Telescope builds used the shared STAR 2.6.1a alignment. SQuIRE was instead run through its native Fetch/Clean/Map/Count pipeline, which performs its own STAR 2.5.3a alignment (--read_length 150, default multi-mapping); because SQuIRE Map aligns with --alignEndsType EndToEnd, reads for SQuIRE were first adapter- and quality-trimmed with Trim Galore 0.6.10 (Krueger *et al*. 2023; Cutadapt 1.18, Martin 2011); paired-end, Phred 20, minimum length 20 bp, Illumina adapter auto-detected. For RSEM quantification, the hg38 refGene annotation and the hg38 RepeatMasker TE annotation were concatenated and filtered to the 192 chromosomes and scaffolds present in the genome FASTA. A transcript reference was extracted from this annotation with rsem-prepare-reference --gtf --star (RSEM 1.2.28, STAR 2.7.11b). Because RSEM requires transcriptome-space alignments, reads were re-aligned with STAR 2.7.11b under RSEM’s ENCODE3 parameter set rather than reusing the shared STAR 2.6.1a genome alignments used elsewhere in this study with (rsem-calculate-expression --star --paired-end --forward-prob 0).

Subfamily-level comparisons used Pearson correlation and DESeq2 differential expression concordance (log2 fold change correlation, significance concordance). Locus-level comparisons used per-locus count ratios [log2(MAJEC/Telescope)] stratified by overlap classification.

#### 2.8.2 Overlap classification

TE loci were classified as intergenic, intronic, or exon-overlapping based on their intersection with annotated gene features. To process the ∼4.7 million annotated TE loci efficiently, classification was performed using a custom, chromosome-parallelized Python script utilizing a binary search algorithm over sorted genomic intervals. A TE locus was first evaluated for overlap with any annotated gene body; loci lacking overlap were classified as intergenic. Genic TEs were subsequently evaluated against exon intervals. To match Telescope’s unstranded quantification model, this exon intersection was performed independent of strand direction. Loci overlapping an exon on either strand were classified as exon-overlapping, while the remaining genic loci were classified as intronic. Of 4.69 million annotated TE loci, 54% were intergenic, 45% intronic, and 1.1% exon-overlapping.

#### 2.8.3 Differential expression comparison

DESeq2 1.38.3 (Love *et al*. 2014) was run independently on MAJEC and Telescope count matrices. For MAJEC, the full count matrix (gene transcripts + TE loci) was provided to DESeq2, with genes contributing to size factor estimation; for Telescope, TE loci only were provided, consistent with each tool’s standard usage. TE loci with ≥5 total counts in ≥2 samples for a given tool were retained for comparison. Significance was assessed at *P*_adj_ < .05.

#### 2.8.4 Vignette analysis

Individual loci exhibiting discordant calls between tools were examined using stranded coverage plots (fractional depth, 1/NH weighting) generated from the BAM files. For the TEtranscripts comparison, DESeq2 was run on TEtranscripts output and compared to MAJEC gene-level and TE locus-level results at loci where MAJEC identified TE-dominated signal (TE log2FC > gene log2FC).

#### 2.8.5 Gene-only (TE-free) simulation

Reads were simulated with polyester 1.38.0 (Frazee *et al*. 2015) exclusively from GENCODE v44 protein-coding and lncRNA transcripts, with no transposable-element sequence present (40 001 333 fragments per sample, two replicates, 150 bp, error rate 0.005). Any read a quantifier assigns to a TE locus is therefore a false positive by construction. Simulated reads were aligned and quantified with the same STAR parameters and MAJEC, released Telescope (v1.0.3, unstranded), and development-branch Telescope (1.0.3 + 48.g4cf1859, stranded) settings as the decitabine benchmark. False-positive rate was computed as the fraction of simulated signal assigned to TE loci, and the residual false positives were broken down by TE family.

#### 2.8.6 Parameter sensitivity and feature ablation

Forty-three parameter conditions were evaluated on the LongBench dataset using MAJEC’s cached mode, which reuses featureCounts assignments and junction metrics across runs (∼14 min per condition, ∼11 h total CPU). Feature ablation removed each prior component individually (completeness penalties, subset penalties, junction boosts, paired rescue, terminal recovery, territory adjustments). Parameter sweeps varied individual parameters across their range while holding others at defaults. Performance was assessed as median log1p fold error against consensus long-read ground truth, with Wilcoxon signed-rank tests comparing each condition to Salmon.

### 2.9 Software and data availability

MAJEC v0.1.0 is implemented in Python and is available at https://github.com/calico/majec under the Apache 2.0 license. All analyses used the following software: STAR 2.7.11b (isoform benchmarks), STAR 2.5.3a (SQuIRE), and 2.6.1a (TE benchmark), Salmon 1.10.3, RSEM 1.2.28, Telescope 1.0.3, TEtranscripts 2.2.3, SQuIRE 0.9.9.92, DESeq2 1.38.3, featureCounts 2.1.1 (Subread), seqtk 1.5-r133, polyester 1.38.0, Trim Galore 0.6.10, Cutadapt 1.18, IsoQuant 3.9.0, and pbmm2 1.9.0. Gene annotations were from GENCODE v44 (LongBench), GENCODE v29 (T-cell), and refGene (Sequins, TE benchmark); TE annotations were from RepeatMasker (hg38). Notebooks and data for figure generation are located at https://doi.org/10.5281/zenodo.19224158. RNA-seq data generated for this study are deposited at SRA PRJNA1454087. LongBench data were obtained from GSE303762. Raw short read Sequins spike-in data were obtained from GSE164598 and sequins GTF, ground truth, and processed longread data are from https://github.com/XueyiDong/LongReadRNA.

## 3 Results

### 3.1 Junction-informed isoform quantification is accurate and mechanistically grounded

The joint gene + TE model depends on accurate assignment of genic reads to their correct transcripts—errors in isoform quantification propagate directly into TE estimates. We therefore first validated MAJEC’s junction-informed EM (Fig. 1a) against established isoform quantification tools using two complementary benchmarks with the goal of demonstrating that the mechanics of MAJEC’s EM and junction priors produce reasonably accurate isoform quantification by comparing it to gold standards Salmon and RSEM. MAJEC need not be (and is not) a markedly better quantifier of isoform expression than these field standards to provide benefits in TE space.

**Figure 1:**
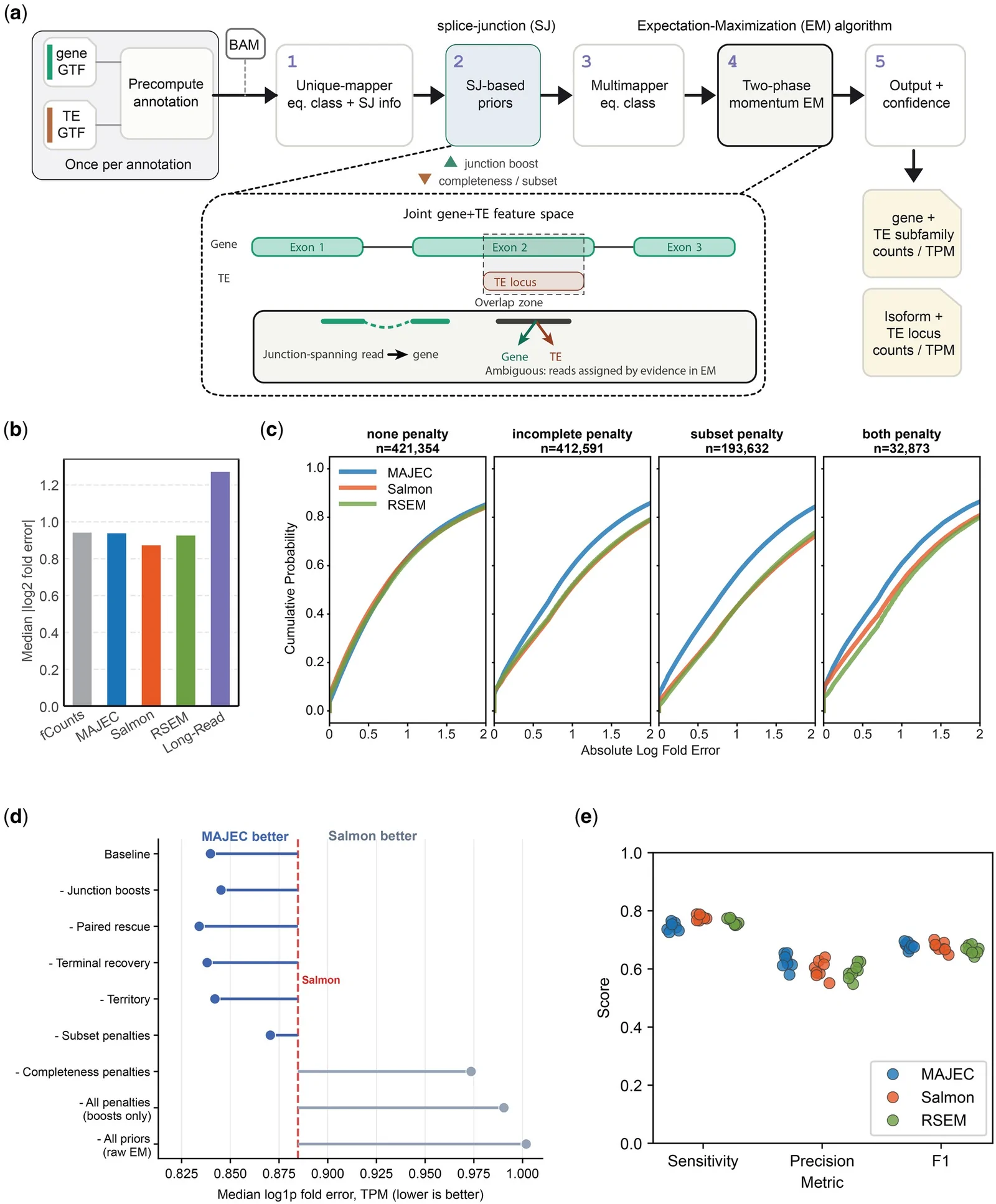
MAJEC isoform quantification benchmarking. (a) Schematic depicting information flow and design of the MAJEC quantification pipeline. From the gene and TE annotations, a shared feature index is precomputed once per annotation (gray). For each sample MAJEC then (i) builds equivalence classes (“eq. class”) for uniquely mapped reads and extracts splice-junction (SJ) evidence from read alignments; (ii) converts that SJ evidence into transcript-level priors, boosting isoforms whose annotated junctions are observed and penalizing those with missing or under-covered junctions; (iii) builds equivalence classes for multi-mapping reads; (iv) resolves all reads with a two-phase, momentum-accelerated expectation–maximization (EM) algorithm that fixes uniquely mapped reads first and then allocates multi-mappers; and (v) reports counts (and TPM) at gene + TE-subfamily and isoform + TE-locus levels with per-feature confidence. Inset: Joint gene + TE feature space. A read falling in an overlap zone (here a TE locus within exon 2) MAJEC lets the two features compete for each such read in the EM, weighted by the SJ-derived priors and other unambiguous evidence. (b) Median log2 fold error against known ground truth concentrations for the Sequins v2.4 spike-in dataset (160 isoforms across 76 genes). (c) CDF of log fold error of MAJEC, Salmon, RSEM against consensus long-read ground truth (mean of dRNA, PacBio Iso-Seq, and ONT cDNA). Pooled transcript measurements from 8 different cell lines are analyzed and stratified by the junction-based penalty assigned by MAJEC (see Section 2). MAJEC performance is best on penalized transcripts. (d) Feature ablation analysis. Each row shows the effect of disabling a single MAJEC feature on median log1p fold error across 8 cell lines relative to the consensus long read data. The dashed red line indicates Salmon’s median error. Subset and completeness penalties contribute the largest individual improvements. “All penalties” removes completeness and subset penalties while retaining junction boosts; “All priors” disables all junction-informed adjustments, leaving only the unweighted EM. (e) Sensitivity, precision, and F1 for each short read quantification against the consensus long-read values. Data from the eight individual cell lines are represented as dots.

On the Sequins v2.4 synthetic spike-in dataset (160 isoforms across 76 genes with known ground truth concentrations), MAJEC, Salmon, RSEM, and raw featureCounts fractional counting all achieved similar accuracy, with median absolute log2 fold errors of 0.88–0.95 (Fig. 1b, Fig. 1, available as supplementary data at *Bioinformatics* online). No method was meaningfully better or worse on this simple transcriptome (max 4 isoforms per gene) where all annotated transcripts are explicitly present, confirming that MAJEC’s junction priors do not introduce systematic bias when they have little work to do.

We next evaluated MAJEC’s performance on complex real transcriptomes using eight ENCODE lung cancer cell lines with matched short-read and long-read data (LongBench). Pooling across cell lines and long-read ground truths (∼1 million transcript-level comparisons), MAJEC produced similar overall quantification error as Salmon (lower on 54.6% of transcripts, median Δ = −0.062) and RSEM (54.5%, median Δ = −0.052). The finding was consistent across all eight cell lines and all four long-read ground truths (dRNA, PacBio Iso-Seq, ONT cDNA, and their consensus average), with the dRNA platform showing the largest overall difference (56.0%, Δ = −0.098 MAJEC versus Salmon). MAJEC demonstrated modest improvements in Pearson correlation across all cell lines and ground truths (Fig. 2, available as supplementary data at *Bioinformatics* online).

**Figure 2:**
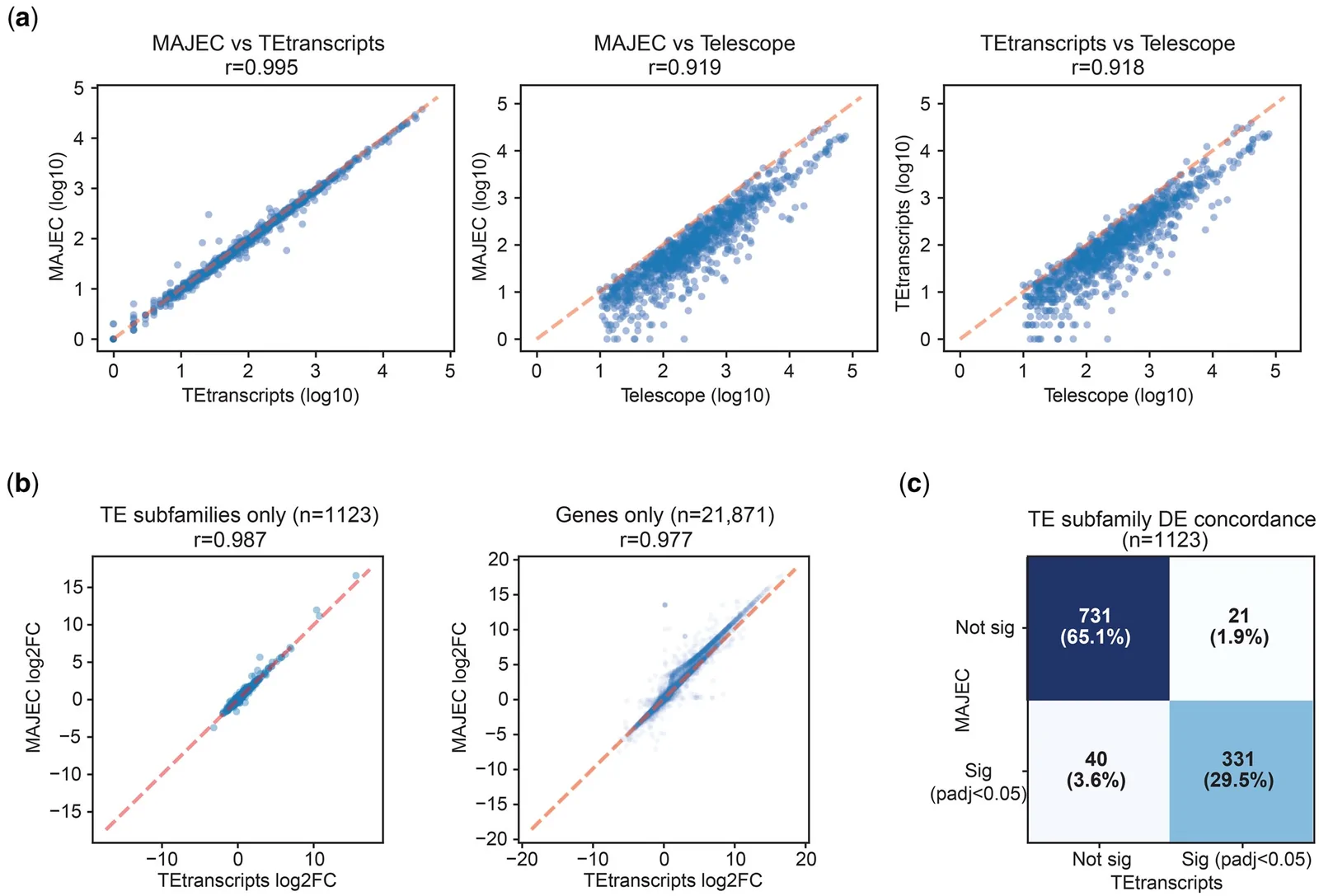
Mechanistically distinct gene-aware TE quantification platforms agree and contrast with TE-only feature space quantification. (a) Pair-wise scatter plots showing the agreement of subfamily TE counts from MAJEC, TEtranscripts and Telescope in Log 10 count space. One replicate of the decitabine treated cells are shown but are highly representative of general results. MAJEC and TEtranscripts demonstrate very strong agreement while both similarly diverge from the gene-unaware Telescope data. (b) Scatter plots showing Log2 fold change concordance between MAJEC and TEtranscripts for TE subfamilies and genes. (c) Concordance matrix showing strong agreement in significance calls by MAJEC and TEtranscripts on subfamilies.

To better understand the performance of MAJEC’s penalty system, we stratified transcripts based on the two junction-based priors MAJEC computes prior to the EM: a completeness penalty (reflecting whether expected splice junctions were missing) and a subset penalty (reflecting whether the transcript’s junction evidence is better explained by an overlapping isoform). Transcripts can receive either penalty, both, or neither (Fig. 3, available as supplementary data at *Bioinformatics* online). Transcripts with strong, isoform-specific junction support also receive a positive boost to their initial estimates.

**Figure 3:**
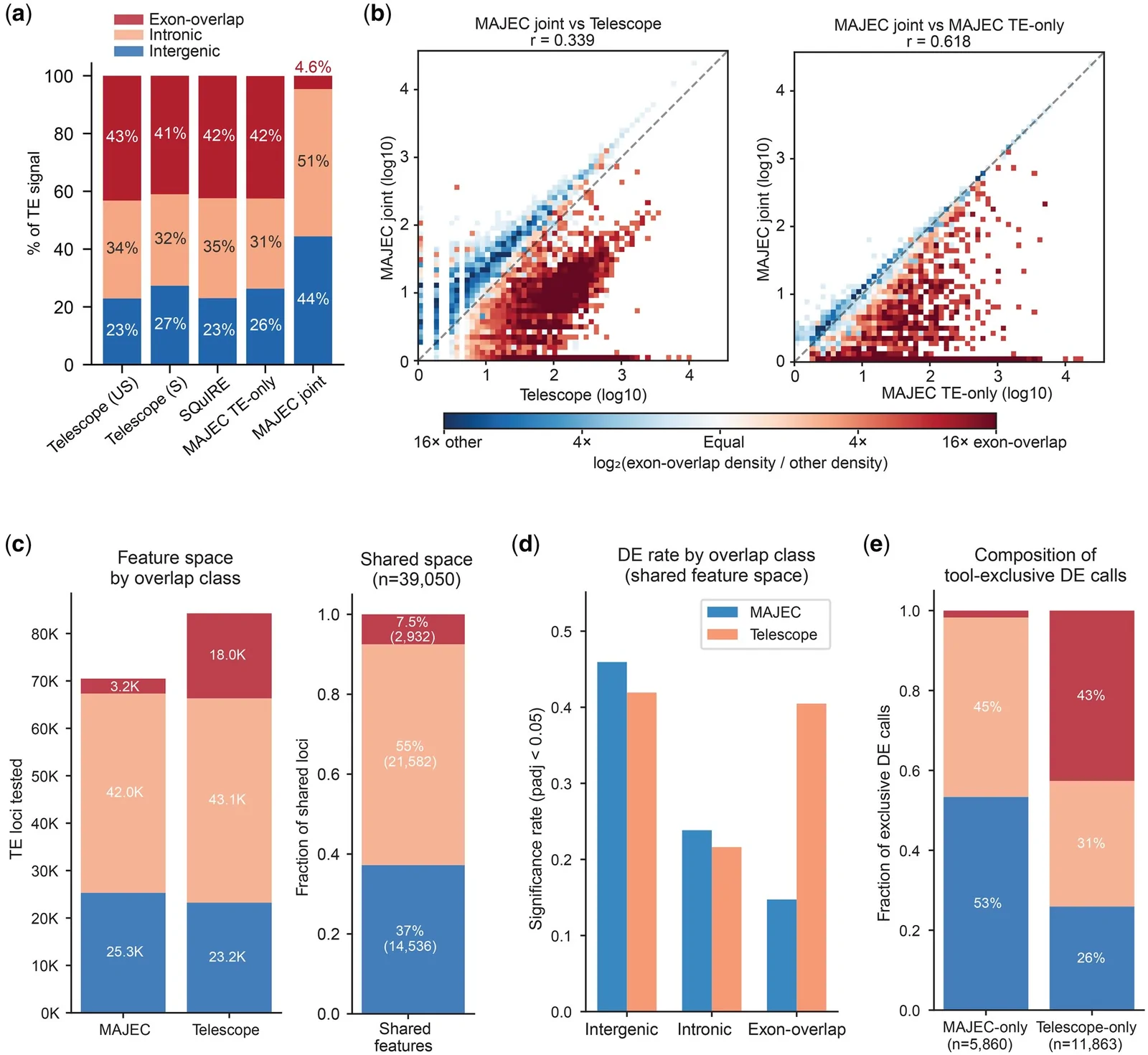
Joint gene-TE modeling eliminates exon-overlap contamination in locus-level TE quantification. (a) Fraction of total TE signal (counts) by genomic overlap class: intergenic (not within any annotated gene), intronic (within a gene body but not overlapping an annotated exon), or exon-overlapping (overlapping an annotated exon on either strand). Values are for five TE-quantification methods on a single library, with all loci classified against the same refGene gene model. Telescope (US) is unstranded; Telescope (S) is stranded (--stranded_mode RF, v1.0.3). The four TE-only methods attribute >40% of TE signal to exonoverlapping loci, including SQuIRE despite its use of a gene annotation; MAJEC joint reduces this to 4.6%. (b) Density scatter plots of per-locus counts between indicated tools, colored by local enrichment of exonoverlapping versus non-overlapping TE loci. Red indicates exon-overlap enrichment; blue indicates intergenic/intronic enrichment. Points below the diagonal represent exon-overlapping loci where Telescope or MAJEC TE-only assigns substantially more signal than MAJEC joint. (c) Left: total TE loci passing count thresholds for DESeq2 analysis, by overlap class. Right: overlap class composition of the 39 050 loci in the shared feature space. (d) Proportion of shared-space loci called significant (*P*_adj_ < .05) by each tool, stratified by overlap class. (e) Overlap class composition of DE calls exclusive to each tool (called significant by one tool but not the other, including tool-exclusive feature space).

Stratifying the data by these junction penalty classifications revealed a clear dependence between MAJEC’s performance and structural complexity (Fig. 1c). On transcripts for which MAJEC applies minimal junction-based penalties (∼40% of the transcriptome) the three tools exhibit similar accuracy. Transcripts penalized for incomplete junction coverage (∼39%) showed a modest MAJEC advantage (56.0% win rate, Δ = −0.100; 55.9% versus RSEM, Δ = −0.104), and transcripts penalized as subsets of better-supported isoforms (∼18%) showed the largest improvement (63.9% win rate, Δ = −0.242; 59.8% versus RSEM, Δ = −0.159). Transcripts carrying both penalties (∼3%) behaved like the single-penalty strata (56.4% and 57.6%). This establishes that MAJEC’s junction- based priors are effective in reducing quantification error for these structurally unsupported isoforms: transcripts entering the EM with strongly penalized starting points are correctly identified in penalizing them improved accuracy, while those with unpenalized starting points maintain baseline parity with competing methods. These results generalized well across all individual cell lines and ground truths (Fig. 2, available as supplementary data at *Bioinformatics* online).

Feature ablation on the LongBench dataset confirmed this interpretation from the opposite direction (Fig. 1d). Removing completeness penalties—the largest single contributor—caused MAJEC to lose to Salmon by approximately 10% in median error, while removing subset penalties increased MAJEC error. Removing both penalty types further degrade model performance, demonstrating that the core innovation is penalizing implausible transcript assignments rather than boosting plausible ones. The remaining features (paired rescue, terminal recovery, territory adjustments) had marginal global impact on this dataset (Fig. 4, available as supplementary data at *Bioinformatics* online).

**Figure 4:**
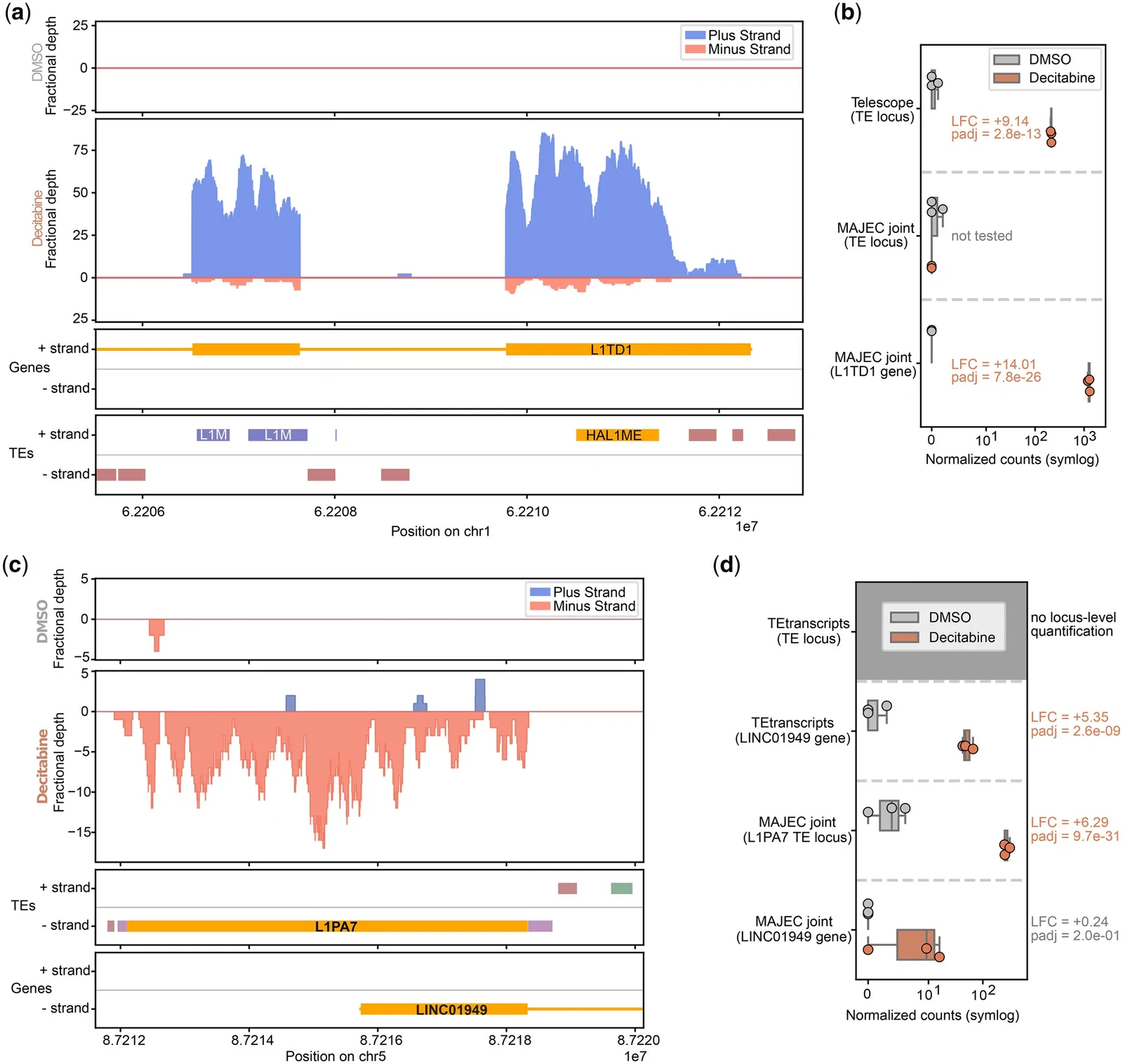
Complementary failure modes of gene-unaware and rule-based TE quantification. (a) Stranded fractional read coverage at the 3′ end of L1TD1 (chr1) in DMSO (top) and decitabine (middle) conditions, with gene and TE annotation tracks below. Plus-strand coverage in decitabine shows a clear exon-intron pattern matching L1TD1; no independent signal is visible over the HAL1ME or L1MEd annotations. (b) Normalized counts (symlog scale) for the HAL1ME locus from Telescope and MAJEC, and for the L1TD1 gene from MAJEC. Telescope reports HAL1ME as highly significantly upregulated (LFC = +9.14, *P*_adj_ = 2.8 × 10^–13^); MAJEC assigns insufficient counts to test the TE locus and instead reports massive upregulation of L1TD1 (LFC = +14.01, *P*_adj_ = 7.8 × 10^–26^). (c) Stranded fractional read coverage at LINC01949 (chr5), showing diffuse minus-strand coverage spanning the L1PA7 element in decitabine without splice structure matching the LINC01949 annotation. (d) Normalized counts for the L1PA7 locus and LINC01949 gene from TEtranscripts and MAJEC. TEtranscripts lacks locus-level TE resolution (gray, top row) and misattributes L1PA7-derived signal to LINC01949 (LFC = +5.35, *P*_adj_ = 2.6 × 10^–9^). MAJEC assigns signal primarily to the L1PA7 locus (LFC = +6.29, *P*_adj_ = 9.7 × 10^–31^) and reports LINC01949 as unchanged (LFC = +0.24, *P*_adj_ = .20).

To further explore, what drives the modest difference between MAJEC and Salmon, we tested whether intrinsic transcript properties predict which tool is more accurate. Restricting to protein-coding and lncRNA transcripts (96% of the annotated set; the biotypes reliably measured by long-read sequencing), we modeled per-transcript win/loss as a function of isoform count, exon count, spliced length, GC content, and expression in a standardized multivariable logistic regression [*n* = 1 053 700 (1 001 718 decided)] transcript–sample observations, (S5A). MAJEC’s advantage concentrated on structurally complex, low-abundance transcripts: the win rate rose with isoform count (41.1% for single-isoform genes to 57.9% for genes with ≥6 isoforms; OR 1.17 per standard deviation [SD]), peaked at intermediate exon counts (58.9% at 4–6 exons; OR 1.08 per SD), and was highest at low expression (60%–63% for Q1-3, falling to 46.3% in the highest; OR 0.946 per SD; Fig. 5, available as supplementary data at *Bioinformatics* online). The performance of MAJEC is thus relatively strongest in the regimes in which read-to-isoform assignment is most ambiguous and junction-informed priors are most informative. Conversely, where there is less structural ambiguity to resolve, MAJEC underperformed: single-isoform genes (41.1%) and single-exon transcripts (42.3%; S5B). Salmon’s clearest advantage was on longer, more highly expressed transcripts (spliced length OR 0.79 per SD), where read evidence is abundant and assignment is largely unambiguous. GC content had a small but significant effect on the outcome (OR 0.98).

At the transcript detection level, MAJEC traded a small amount of sensitivity for slightly improved precision (Fig. 1e). Compared to Salmon, MAJEC detected fewer true transcripts (sensitivity 0.745 versus 0.777) but called 3800–6900 fewer false positives per cell line (mean 5800; precision 0.626 versus 0.599), resulting in a similar composite F1 score (0.680 versus 0.676 for Salmon; 0.666 for RSEM). This slight tilt towards precision is directly relevant to the joint gene + TE model: suppressing unsupported gene transcripts prevents false genic features from competing with TE loci for shared reads during the EM.

These findings replicated on an independent T-cell dataset, where MAJEC showed the same penalty-type-dependent performance versus Salmon and RSEM: against Salmon, 50.3% on unpenalized transcripts rising to 57.5% on incomplete-penalized and 71.6% on subset-penalized transcripts; against RSEM, 46.4%, 51.3%, and 56.5% across the same classes. Full results in (Fig. 6, available as supplementary data at *Bioinformatics* online).

To examine how sequencing depth affects accuracy for MAJEC, we downsampled the short reads of three LongBench cell lines to 7.5, 15, 30, and 55 M read pairs per line and re-quantified each subset with MAJEC and Salmon. Isoforms were stratified by their long-read intra-gene structure into dominant, minor, and rare classes (rare = minor isoforms contributing <5% of their gene’s long-read signal), and median log-fold error and detection sensitivity were evaluated on the long-read–confirmed isoforms in each class (Fig. 7, available as supplementary data at *Bioinformatics* online). Both tools improved in accuracy with depth. MAJEC’s advantage was concentrated in the rare class: its median error was lower than Salmon at every depth (1.024 → 0.843 from 7.5 to 55 M, versus Salmon 1.126 → 0.965), with lower error in all three cell lines at every depth. The two tools also differed sharply in the depth dependence of rare-isoform detection: MAJEC’s sensitivity was essentially flat across the range (0.72–0.75), whereas Salmon’s rose steeply from 0.551 at 7.5 M, drawing level at 55 M (0.748 versus 0.752). The two higher-abundance classes behaved differently from each other: in the dominant class MAJEC retained a median error edge at every depth (0.563 → 0.482 versus Salmon 0.581 → 0.514), whereas in the minor class its early advantage (0.802 versus 0.935 at 7.5 M) was erased by 30 M and reversed by 55 M (0.739 versus 0.698). MAJEC thus shows no tool-specific, disproportionate degradation on low-coverage data, and is if anything comparatively more sensitive and accurate than Salmon when reads are sparse.

Long-read quantification serves as an approximate rather than absolute ground truth, and itself showed the highest error on the Sequins benchmark (median 1.28 versus 0.88–0.95 for short-read methods). However, several features of the LongBench results argue against MAJEC’s performance on penalized transcripts as being an artifact of ground truth bias: the difference was consistent across three independent long-read platforms with distinct error profiles (dRNA, PacBio Iso-Seq, ONT cDNA), was mechanistically confined to transcripts receiving strong junction penalties, and was eliminated by feature ablation. Together, the two benchmarks support the aim of this section: Sequins establishes accuracy against absolute ground truth on simple genes, LongBench shows that accuracy holds on complex isoform structures and both place MAJEC on par with dedicated isoform quantifiers, the prerequisite for the joint gene–TE applications that follow.

This performance comes with identifiable limitations. On the Sequins dataset, asymmetric isoform pairs with highly imbalanced unique evidence can trigger over-concentration, where the EM iteratively assigns all shared reads to the dominant isoform, e.g. R2_7_1 (Fig. 1, available as supplementary data at *Bioinformatics* online). MAJEC’s performance is strongest at low expression (<3 CPM, 60.6% win rate versus Salmon), where junction penalties effectively suppress marginal transcripts. At moderate expression levels (10–100 CPM), Salmon’s sequence-level modeling—including GC and fragment-length bias correction that MAJEC does not implement—provides an edge (win rates 44%–45%). The two methods converge at higher expression (>100 CPM), where both have abundant evidence and the modeling differences matter less (Fig. 2c, available as supplementary data at *Bioinformatics* online).

### 3.2 MAJEC produces TEtranscripts-equivalent subfamily quantification

Having established the reliability of MAJEC’s isoform quantification, we next evaluated its TE subfamily estimates against the most widely used TE quantification tool, TEtranscripts, and the locus-resolution tool Telescope. To evaluate TE quantification in a biologically relevant context, we generated stranded 150 bp paired-end RNA-seq from FaDu head and neck squamous cell carcinoma (HNSCC) cells treated with DMSO or 1 µM decitabine for 7-days in triplicate. Decitabine, a DNA methyltransferase inhibitor, induces widespread TE demethylation and reactivation alongside gene-level expression changes, providing a dataset where both gene and TE differential expression are expected.

At the subfamily level, MAJEC and TEtranscripts produced near-identical count estimates (Pearson *r* = 0.993–0.995 across samples; Fig. 2a, left panel), with strong agreement across all major TE classes (LINE, SINE, LTR, DNA; *r* = 0.990–0.999; Fig. 8a, available as supplementary data at *Bioinformatics* online). This agreement is notable because the two tools use fundamentally different EM architectures: TEtranscripts distributes multi-mapped reads across TE subfamilies directly, while MAJEC resolves reads to individual TE loci and then aggregates to the subfamily level. The convergence of these two approaches on the same subfamily totals provides independent validation of both.

Both MAJEC and TEtranscripts diverged from Telescope by a similar magnitude (MAJEC–Telescope *r* = 0.919; TEtranscripts–Telescope *r* = 0.918; Fig. 2a, center and right panels). This symmetric pattern is informative. MAJEC and Telescope use more similar EM architecture—locus-level resolution followed by aggregation—yet MAJEC agrees far more closely with TEtranscripts than with Telescope. The variable that distinguishes the grouping is not the EM granularity but how each tool handles genic contamination. MAJEC addresses it dynamically through its joint gene + TE EM model, where genes and TE loci compete for reads probabilistically. TEtranscripts addresses it through a different mechanism: a hard-coded prioritization rule that assigns uniquely mapped reads overlapping both a gene exon and a TE annotation exclusively to the gene. Telescope, by contrast, operates entirely blind to the gene feature space and retains genic reads with no mechanism to redistribute them. A third gene-aware model, RSEM run on the combined gene + TE tests whether the gene-aware/gene-unaware split holds at the locus level. It does, with one structural caveat: RSEM’s per-sample effective-length model assigns zero effective length to ∼36% of the 4.53 M TE loci (median length 230 bp; 66.6% under 300 bp; Fig. 9a, available as supplementary data at *Bioinformatics* online), excluding >42 000 loci that carry ≥1 MAJEC count and account for 13.1% of MAJEC’s total TE signal. On the loci RSEM can quantify, MAJEC and RSEM were more concordant with each other than either was with Telescope at every minimum-length cutoff (*r* = 0.92 at ≥500 bp versus 0.66–0.71 for both Telescope pairs; Fig. 9b, available as supplementary data at *Bioinformatics* online). Gene-aware methods thus converge on the same estimates through three independent strategies—a probabilistic joint model, a rule-based heuristic, and a transcriptome wide isoform EM—while the only gene-unaware tool stands apart. This convergence is among the strongest evidence that the joint model is producing correct TE estimates rather than merely different ones.

MAJEC’s EM also improved subfamily estimates relative to the pre-EM fractional counts. The mean absolute log2 fold change between MAJEC and TEtranscripts decreased from 0.40 (pre-EM) to 0.19 (post-EM), indicating that the EM redistributes multi-mapped reads in a manner consistent with TEtranscripts’ subfamily-level model. Notably, the EM’s effect on subfamily estimates was nearly unidirectional, reducing counts as genic reads were reassigned from TE subfamilies to overlapping gene features (Fig. 8b, available as supplementary data at *Bioinformatics* online).

At the differential expression level, MAJEC and TEtranscripts showed high concordance. DESeq2 log2 fold change estimates correlated at *r* = 0.977 overall and *r* = 0.987 for TE subfamilies specifically (Fig. 2b). Of TE subfamilies called as significantly differentially expressed by either tool, 94.6% were concordant in direction and significance, while the 61 discordant subfamilies (5.4%) still demonstrated strong log2fold correlation (*r* = 0.88) (Fig. 2c and Fig. 8c, available as supplementary data at *Bioinformatics* online). MAJEC can therefore serve as a drop-in replacement for TEtranscripts in existing DE workflows, with the additional benefit of locus-level resolution that TEtranscripts does not provide.

### 3.3 Joint gene–TE modeling eliminates false TE signal from genic loci

The central motivation for MAJEC’s joint model is the pervasive overlap between TE annotations and protein-coding genes. Approximately 45% of the human genome derives from TEs, and many TE loci reside within gene bodies—in introns, UTRs, and occasionally exons. When a gene is transcribed, reads originating from the gene’s mRNA will overlap these embedded TE annotations. A TE-only quantification model has no mechanism to distinguish such genic reads from reads produced by independent TE transcription, leading to systematic false TE signal at gene-overlapping loci.

To quantify this problem, we classified TE loci by their relationship to gene annotations—ignoring strand, since Telescope’s released version does not model strandedness—as intergenic (54.2% of loci), intronic (44.7%), or exon-overlapping (1.1%), and measured the fraction of total TE signal originating from each class.

The results revealed exonic overlap to be a dominant source of contamination. In Telescope, exon-overlapping TE loci accounted for 43% of total TE signal (876K of 2.03M fragments; Fig. 3a). This was not issue of strandedness as the unreleased, development, version of Telescope which has a stranded mode left the pattern essentially unchanged (41% exon-overlapping). Moreover, MAJEC run in a TE-only mode (identical EM but without gene features) reproduced it closely (42%) and the same contamination arose in SQuIRE (42%), a tool that *does* incorporate a gene annotation, but quantifies TE loci in a separate feature space. Only MAJEC’s joint model, in which genes and TE loci compete for each read within a single EM, reduced exon-overlapping signal to 5% (Fig. 3a). RSEM on the combined gene + TE reference likewise kept exon-overlapping signal low (7.5% versus 4.7% for MAJEC among loci ≥300 nt; Fig. 9c, available as supplementary data at *Bioinformatics* online), an independent, second-method confirmation that joint gene–TE modeling resolves this contamination.

Because Fig. 3a is based on real data, exon-overlapping TE signal could in principle include genuine TE transcription rather than contamination. To measure specificity directly, we simulated 40 million paired-end fragments from GENCODE coding and lncRNA transcripts only, with no TE sequence present, so any TE assignment is a false positive (Fig. 10, available as supplementary data at *Bioinformatics* online). MAJEC misassigned 0.11% of fragments to TEs, compared with 8.0% for released (unstranded) Telescope and 4.2% for a development stranded build (75-fold and 40-fold higher misattribution). Strand-awareness roughly halved Telescope’s rate but did not change the composition of its errors, which were concentrated in ancient, gene-embedded Alu, MIR, and L2 elements. MAJEC’s smaller residual was instead dominated by young L1 subfamilies (L1HS and L1PA, 58%), likely reflecting cases where L1 sequence makes up a large enough fraction of the source transcripts that the reads cannot be confidently reassigned to a non-TE feature.

Exonic overlap was a major contributor to differences in expression estimates between the MAJEC joint model and Telescope or MAJEC TE-only mode (Fig. 3b). The joint model’s effect is confined to exon-overlapping loci. Comparing MAJEC joint to MAJEC TE-only, intergenic and intronic loci are virtually unchanged (*r* = 0.99 and 0.96, median log2FC = 0.00 for both), while exon-overlapping loci are sharply reduced (median log2FC = −2.32; Fig. 11a, available as supplementary data at *Bioinformatics* online). Against Telescope, the differences are larger across all classes (intergenic *r* = 0.52, intronic *r* = 0.39, exon-overlapping *r* = 0.34; Fig. 11b, available as supplementary data at *Bioinformatics* online), reflecting additional algorithmic distinctions between the tools—strand awareness, EM priors, initial assignments—beyond the gene model itself. Intergenic signal increased under the joint model (464K to 639K fragments, median log2 ratio = +0.42), consistent with multi-mapper redistribution from deflated exonic copies to intergenic loci where no gene competes.

Exon-overlap contamination is therefore an architectural property of quantifying TEs outside a joint gene–TE feature space, shared by gene-unaware (Telescope), TE-only (MAJEC TE-only), and even gene-annotation-using (SQuIRE) methods alike.

The contamination flows through to differential expression analysis. We performed DESeq2 comparing the DMSO and decitabine treated cells on TE features having at least 5 counts in 2 or more samples. The number of features was generally similar between MAJEC (71K) and Telescope (84K) with Telescope having an enrichment in exon-overlapping TE consistent with the count analyses above (Fig. 3c). There were 39,050 TE loci shared between MAJEC and Telescope, and in that overlapping feature space the two methods detected similar overall numbers of significant DE loci (MAJEC: 12 256; Telescope: 11,948; *P*_adj_ < .05) but with substantially differing composition (Fig. 3c and d). Telescope called nearly three times more exon-overlapping loci as significant (1187 versus 432, or 40.5% versus 14.7% of shared exon-overlap loci), while MAJEC detected more significant intergenic (6678 versus 6095) and intronic (5146 versus 4666) loci (Fig. 3d, Fig. 12a, available as supplementary data at *Bioinformatics* online).

An additional 45 215 TE loci passed the count threshold in Telescope but not MAJEC; Telescope called 9991 of these as significant, with exon-overlapping loci showing the highest rate (28.1% significant versus 14.3% for intronic). In total, Telescope produced approximately 12 000 DE calls unsupported by MAJEC, with exon-overlapping loci disproportionately represented. Conversely, MAJEC detected 3680 significant DE calls at 31 457 loci that fell below the count threshold in Telescope (Fig. 12b, available as supplementary data at *Bioinformatics* online). These were overwhelmingly intergenic (58%) and intronic (41%), with only 254 exon-overlapping loci (0.8%)—consistent with multi-mapper redistribution directing signal toward intergenic and intronic loci where autonomous TE transcription is more plausible. In contrast to Telescope’s exclusive feature space, the DE calls private to MAJEC were relatively modest in significance, reflecting low-count loci that gained marginal signal through multi-mapper redistribution rather than large-effect reactivation events (Fig. 13, available as supplementary data at *Bioinformatics* online). To illustrate the practical consequences at individual loci, we examined two vignettes from the same decitabine experiment that together demonstrate the complementary failure modes of existing tools (Fig. 4).

### 3.4 Vignette 1: false TE reactivation (Telescope’s failure)

HAL1ME_dup104 is an element residing within the second exon of L1TD1, a pluripotency-associated gene silenced by DNA methylation in most somatic cells (Altenberger *et al*. 2017, Kavaklioglu *et al*. 2025). Telescope reports this TE locus as highly significantly upregulated by decitabine treatment (LFC = +9.1, adjusted *P* = 2.8 × 10⁻¹³; Fig. 4a and b). In isolation, this suggests the demethylation-induced reactivation of an ancient LINE element. However, MAJEC’s joint model correctly assigns these reads to L1TD1, which shows massive upregulation (LFC = +14.0, adjusted *P* = 7.8 × 10⁻²⁶). Because L1TD1's transcript model has strong junction support across all decitabine samples, it receives a pre-EM junction boost, allowing MAJEC to recognize the signal as unambiguously genic. Stranded coverage confirms this: decitabine-treated samples show strong plus-strand coverage with a clear exon-intron pattern matching L1TD1, with no isolated peak over the TE annotation (Fig. 4a and b). Similar patterns were consistently observed in exon overlapping TE of other decitabine derepressed genes such as IL11 (Fig. 14, available as supplementary data at *Bioinformatics* online).

### 3.5 Vignette 2: false gene upregulation (TEtranscripts’ failure)

The converse error occurs when a genuinely reactivated TE is embedded within a gene. L1PA7_dup4347 is a minus-strand L1PA7 element residing within LINC01949, a noncoding RNA. MAJEC’s joint model correctly separated the two signals: the TE locus was strongly upregulated (LFC = +6.29, *P*_adj_ = 9.7 × 10⁻³¹), while LINC01949 showed no change (LFC = +0.24, *P*_adj_ = .20; Fig. 4c and d). Notably, LINC01949 has two annotated splice junctions but zero junction-spanning reads in any sample, triggering a completeness penalty that reduced its prior—the same junction system that assigns reads to L1TD1 in vignette 1 withholds them from LINC01949. Stranded coverage confirmed genuine TE transcription: minus-strand signal in decitabine samples spanned the L1PA7 annotation without exon-intron structure, consistent with cryptic transcription from a demethylated TE promoter rather than spliced lncRNA expression (Fig. 4c). Because TEtranscripts assigns uniquely mapped reads at gene-TE overlaps exclusively to the gene, these TE-derived reads were absorbed into the LINC01949 count, producing a false DE call (LFC = +5.35, *P*_adj_ = 2.6 × 10^−9^). A researcher using TEtranscripts would conclude they had discovered a decitabine-responsive lncRNA; the real biology was an embedded L1 element being demethylated.

These two vignettes illustrate opposing failure modes that MAJEC’s joint model resolves simultaneously. Telescope, operating without gene annotations, misattributes genic reads to overlapping TEs, while TEtranscripts, applying a rigid gene-over-TE prioritization rule, absorbs genuine TE signal into host genes. The Telescope-direction error is pervasive affecting multiple TE per upregulated gene (Fig. 4a, Fig. 14, available as supplementary data at *Bioinformatics* online). The TEtranscripts-direction error is much less common but systematically biased. Of 52 loci where MAJEC identified TE-dominated signal at a genic locus, TEtranscripts reported significant gene upregulation of the overlapping host gene at 18 (Table 1, available as supplementary data at *Bioinformatics* online). Among the eight host genes with the largest inflated log-fold-change calls, seven were lncRNAs, and the implicated TE families (LTR1A2, LTR12C, HERVH-int, L1PA7, L1PB1, HAL1) include several with documented reactivation or cryptic-promoter activity upon DNA demethylation (e.g. LTR12C; Brocks *et al*. 2017, Chuong *et al*. 2017) (Table 1, available as supplementary data at *Bioinformatics* online).

**Table 1:** Comparative benchmarking of TE tools on runtime and memory.^a^

Tool	Cores	Wall time	Core-hours	Peak RAM (GB)	TE resolution	Gene model
MAJEC^b^	6	20 min	2	52	Locus	Joint
MAJEC (cached)^c^	6	5 min	0.5	52	Locus	Joint
TEtranscripts^d^	1	4 h 55 min	4.9	6/BAM	Subfamily	Joint
Telescope^e^	1 (×6)	30 min/sample	3	13/BAM	Locus	None

a Computational performance and feature comparison of TE quantification tools. Runtime, memory, and model characteristics for processing six BAM files on an Intel Xeon HPC cluster. MAJEC utilizes internal multiprocessing to achieve rapid locus-level, joint gene-TE quantification (20 min wall time, 2.0 core-hours). Its caching mechanism further reduces re-analysis time to 5 min by bypassing initial BAM parsing. TEtranscripts relies on single-threaded execution, requiring 4 h 55 min for subfamily-level resolution. Telescope processes samples individually (∼30 min/sample) but operates in a TE-only feature space. Peak RAM is reported for the joint run (MAJEC) or per individual BAM file (TEtranscripts, Telescope).

b MAJEC v0.1.0 processes all samples jointly with internal multiprocessing (6 worker threads).

c “Cached” denotes re-analysis with modified parameters, bypassing BAM parsing and feature counting.

d TEtranscripts v2.2.3 is inherently single-threaded for the counting and EM steps and not designed for single BAM runs complicating parallelization.

e Telescope v1.0.3 processes one sample per invocation and is trivially parallelizable; wall time per sample ranged from 28 to 33 min across the six samples. Core-hours for Telescope reflects the total across all six samples (6 samples × 30 min × 1 core = 3.0 core-hours).

MAJEC resolves both cases correctly because it does not impose a fixed rule about which feature type prevails. Instead, genes and TE loci compete for reads probabilistically within the joint EM, and the assignment follows the evidence. When the evidence supports genic origin (L1TD1: exon-intron structure, strand-concordant coverage), reads flow to the gene. When the evidence supports TE origin (LINC01949/L1PA7: localized TE-region coverage without splice structure, silent host gene), reads remain with the TE. A probabilistic model gets both cases right because it asks “what does the data support?” rather than applying a predetermined answer.

### 3.6 Runtime and computational requirements

MAJEC provides practical computational efficiency alongside its TE quantification accuracy improvements. We compared the peak memory usage and runtimes from MAJEC, TEtranscripts, and Telescope for our decitabine dataset (Table 1). Both TEtranscripts and Telescope process samples individually on single cores, but their computational bottlenecks differ significantly. Telescope is trivially parallelizable across samples, requiring approximately 31 min to process a single BAM. However, its ∼13 GB per-sample memory footprint means that running six samples simultaneously demands ∼78 GB of RAM, effectively exceeding the capacity of a standard 64 GB workstation. Conversely, TEtranscripts is highly memory efficient (∼6 GB per sample), likely owing to its collapse of the TE feature space to the subfamily level. Yet, because it is inherently designed for differential expression, it requires at least one test and one control BAM and processes them serially at roughly 1 h per sample. This dictates a minimum runtime of 2 h even in a parallelized workflow.

In stark contrast, MAJEC overcomes both of these limitations. Its multithreaded architecture processes all six samples jointly in just 20 min across 6 cores, utilizing a single, shared memory footprint of ∼50 GB. MAJEC’s efficiency advantage is thus roughly 4× in core-hours compared to the combination of Telescope and TEtranscripts needed to approximate its output—while also providing additional wall-time benefits and isoform-level quantification that neither competing tool offers.

Runtime is dominated by I/O rather than computation (Table 2, available as supplementary data at *Bioinformatics* online). The momentum-accelerated EM completed in less than 1.5 min across 15–16 iterations, accounting for less than 10% of wall time. Peak memory (∼50 GB) is within the range of a standard STAR alignment; MAJEC does not require HPC infrastructure. A caching mode that stores featureCounts assignments and junction metrics enables rapid parameter exploration (∼5 min per rerun versus ∼20 min for a full run).

## 4 Discussion

MAJEC demonstrates that gene, isoform, and locus-level TE quantification can be unified in a single analysis that is faster, more comprehensive, and more accurate at gene-TE boundaries than running specialized tools in sequence. Three aspects of these results warrant further discussion: the mechanistic basis of MAJEC’s isoform accuracy, the scope and implications of the gene-TE overlap problem, and the practical limitations of the current implementation.

### 4.1 Unified quantification is faster and simpler

The most immediate practical benefit of MAJEC is consolidation. A typical RNA-seq workflow studying TE biology requires STAR alignment (for visualization and QC), Salmon (for isoform quantification), TEtranscripts (for subfamily-level TE DE), and potentially Telescope (for locus-level TE resolution)—four tools with three different annotation formats and incompatible output structures. MAJEC replaces the three downstream tools with a single analysis that operates on the existing BAM. At comparable CPU allocation to TEtranscripts (1 core/BAM), MAJEC completes in approximately 20% of the time, without sacrificing subfamily-level concordance with TEtranscripts (*r* > 0.99) or locus-level resolution.

Beyond quantification, MAJEC provides integrated downstream tools: a queryable SQLite database storing all count estimates, junction evidence, and confidence metrics; automated extraction of DESeq2-ready count matrices at gene, isoform, and TE locus levels; and interactive HTML reports for isoform-level QC and exploratory analysis. For users whose primary interest is isoform resolution rather than TE biology, MAJEC and Salmon are complementary: Salmon’s sequence-level modeling and GC bias correction capture information that MAJEC’s alignment-based approach does not, while MAJEC provides the joint gene + TE model that alignment-free tools structurally cannot. Running both from the same BAM adds only minutes to the analysis.

### 4.2 Junction penalties are the core innovation—and the mechanism is clear

MAJEC’s isoform accuracy is comparable with Salmon and RSEM with gains confined to specific transcript classes. On simple synthetic transcriptomes (Sequins, max 4 isoforms per gene), all methods performed equivalently. On complex real transcriptomes (LongBench, 8 ENCODE cell lines), MAJEC scored better than Salmon on 54% of transcripts, and this difference was concentrated in transcripts receiving incomplete or subset junction penalties; transcripts with no junction penalty showed no difference between methods. This stratification is consistent with the junction prior contributing where isoform structure is ambiguous and remaining inert elsewhere.

Feature ablation confirmed this interpretation from the causal direction. Removing completeness penalties significantly degraded MAJEC accuracy resulting in performance that was inferior to Salmon by approximately 10% while disabling the subset penalties caused further erosion. MAJEC’s strength is not better evidence for well-supported transcripts; it is better use of evidence to downweigh poorly-supported ones.

The penalty-type stratification results replicate across two independent datasets (T-cell and LongBench), and the effect is consistent across all four long-read ground truths (dRNA, PacBio, ONT, and their consensus), with dRNA—arguably the least biased long-read platform due to the absence of reverse transcription and PCR amplification (Garalde *et al*. 2018, Workman *et al*. 2019)—being the most pronounced.

The precision-sensitivity tradeoff inherent in the penalty system is an intentional design choice. By aggressively penalizing unsupported isoforms, MAJEC calls 3800–6900 fewer false-positive transcripts per cell line than Salmon. This precision is essential for the joint gene + TE model, as it prevents spurious genic transcripts from inappropriately absorbing reads that belong to legitimate TE loci. However, this comes at the cost of suppressing some genuinely expressed, low-abundance transcripts. Users focused purely on discovering rare, novel isoforms may prefer Salmon’s higher sensitivity, while those analyzing TE biology will benefit from MAJEC’s strict genic boundaries. Implementing an expression-dependent penalty floor to reduce penalty strength when total evidence is sparse represents a natural future improvement.

### 4.3 The gene-TE overlap problem is larger than appreciated

The most striking finding of this study is the sheer scale of exonic overlap contamination in locus-level TE quantification. When using Telescope, exon-overlapping loci—which constitute only 1.1% of annotated TE loci—account for 43% of the total TE signal. MAJEC’s joint model reduces this exonic TE signal to just 5% and successfully relocates multi-mapped reads from gene bodies to intergenic loci where autonomous TE transcription is biologically plausible. Because these fractions come from cellular RNA, where a portion of the exon-overlapping signal could in principle be genuine, we turned to a ground-truth control in which RNA-seq was simulated from gene models with no TE transcribed. In that setting MAJEC attributed almost none of the gene-derived signal to TEs (0.11%), nearly two orders of magnitude below Telescope, suggesting that most exon-overlapping TE signal reflects contamination rather than authentic TE expression (Fig. 10, available as supplementary data at *Bioinformatics* online). RSEM run on a combined gene + TE reference corroborates this conclusion: the two independent gene-aware models converge on low exon-overlap signal and high mutual concordance among the loci both can quantify, while RSEM’s effective-length normalization excludes the short-locus space that MAJEC extends into (Fig. 9, available as supplementary data at *Bioinformatics* online).

This contamination severely confounds differential expression analysis. Despite testing in the same DESeq2 framework, Telescope called nearly 12 times more exon-overlapping loci as significantly differentially expressed compared to MAJEC (5415 versus 461). This massive exonic inflation accounts for over 80% of the total discrepancy in DE calls between the two tools. This contamination acts as a double-edged sword: inflated exonic counts artificially generate false positives while simultaneously attracting multi-mapped reads away from intergenic loci during the EM, reducing the statistical power to detect genuine TE activation.

Genic contamination is not a Telescope-specific artifact but a general property of TE-only quantification. We observed the same effect with SQuIRE, which attributes >40% of its TE signal to the exon-overlapping class despite being gene-aware. While SQuIRE ingests a gene annotation, it is only used to quantify genes and to guide splice-aware alignment not to allow arbitration of reads among overlapping gene and TE annotations. Telescope, likewise, was designed to solve the multi-mapper reassignment problem, and does so effectively through its Bayesian model, but that model resolves ambiguity among competing TE loci, not between TEs and overlapping genes. Neither tool performs gene-versus-TE read arbitration and the scale of this contamination was not previously quantified. Our results suggest that published locus-level TE analyses using any TE-only tool should be interpreted with caution at gene-overlapping loci, and that future studies would benefit from either joint gene-TE modeling or, at minimum, *post hoc* filtering of exon-overlapping TE signal.

The overlap problem is also bidirectional. While Telescope and SQuIRE inflate TE signal at loci where genes are expressed, TEtranscripts’ gene-over-TE prioritization rule can absorb genuine TE transcription into host gene counts, creating spurious gene-level differential expression calls. We identified several such cases among 52 loci where MAJEC detected TE-dominated signal at genic loci, where 7 of the 8 most affected host genes were lncRNAs. This systematic bias toward noncoding genes is expected: the majority of lncRNA exonic sequence is TE-derived, making lncRNAs uniquely vulnerable to a rule that unconditionally prioritizes gene annotations (Kelley and Rinn 2012). Several of the affected TE families—notably LTR12C and the LTR1/HERV LTRs—have documented promoter or cryptic-transcription activity that is derepressed upon DNA demethylation (Chuong *et al*. 2017). These are precisely the TEs most likely to be genuinely reactivated by decitabine and, because much lncRNA sequence is TE-derived (Kelley and Rinn 2012), most likely to reside within the noncoding genes susceptible to this misattribution. Studies of TE reactivation in contexts where noncoding transcriptome analysis matters—epigenetic therapy, cancer, development—should be particularly attentive to this class of artifact (Brocks *et al*. 2017).

The convergence between MAJEC and TEtranscripts at the subfamily level, despite fundamentally different approaches to genic contamination, provides strong validation that the joint model produces correct TE estimates. Meanwhile, Telescope diverges from both (*r* ≈ 0.92), confirming that the grouping variable is not the algorithm but whether gene-TE overlap is addressed at all.

### 4.4 Limitations and future directions

Several limitations of the current implementation suggest directions for improvement. The EM can over-concentrate counts for asymmetric isoform pairs where one transcript has substantially more unique evidence than the other, iteratively driving the minor isoform to zero. This affects a small number of transcripts (approximately 1000 across 8 cell lines, identifiable through the discord score) and could be addressed with pseudocount regularization. More fundamentally, the current architecture separates feature assignment from junction evidence: featureCounts assigns reads to equivalence classes based solely on exon overlap, discarding the splice junction information encoded in read CIGAR strings and the per-base alignment quality (mismatch counts and alignment scores) that could distinguish near-identical paralogs and processed pseudogenes from their parent loci before the EM stage. A read spanning two exons connected by a transcript-diagnostic junction is indistinguishable from one overlapping the same exons without splicing. For paired-end libraries, intersecting the transcript sets from both mates of a fragment provides additional narrowing, as a fragment can only originate from a transcript compatible with both ends. A prototype single-pass BAM tool implementing this approach consolidates the current three-pass pipeline (unique mapper assignment, multimapper filtering, multimapper assignment) into a single scan that simultaneously resolves exon overlap, junction compatibility, and read-pair agreement. Incorporating alignment metrics into equivalence-class weighting through a direct BAM parser is a natural extension of the single-pass prototype noted above. Whether tighter equivalence class resolution or incorporation of alignment quality can translate to improved quantification accuracy remains to be validated against long-read ground truth. Parameter sensitivity analysis also identified marginal improvements on the LongBench dataset, though these may be dataset-specific and require cross-validation before changing defaults.

MAJEC is alignment-based and therefore slower than Salmon for pure isoform quantification. However, TE quantification inherently requires genomic alignment—multi-mapped reads to repetitive genomic loci cannot be resolved by alignment-free approaches that operate on transcript-level indices—so the comparison for users needing TE analysis is not MAJEC versus Salmon but MAJEC versus the combined multi-tool pipeline. In this comparison, MAJEC is several times faster.

The joint model’s effectiveness depends on the quality of both gene and TE annotations. On well-annotated genomes (human, mouse) with comprehensive GENCODE transcript models and RepeatMasker TE libraries, the model can confidently assign reads at gene-TE boundaries. For non-model organisms with incomplete gene annotations, the benefit of joint modeling is reduced—poorly annotated genes cannot absorb their genic TE reads. Comparisons between MAJEC and Telescope output were performed without strand discrimination, as Telescope lacks strand awareness; this introduces ambiguity at loci where TEs overlap antisense genes and may account for some disagreements between the two methods.

Finally, long-read sequencing, which increasingly resolves multi-mapping ambiguity through read length alone, represents a complementary approach to the same problems MAJEC addresses computationally; recent tools such as LocusMasterTE (Lee *et al*. 2025) leverage long-read data for improved locus-level resolution. TEspeX (Ansaloni *et al*. 2022) addresses the exonized TE fragment problem through a filtering rather than modeling approach. Both tools recognize the gene-TE overlap problem from different angles, and MAJEC’s probabilistic joint modeling represents a third strategy that is complementary to both.

## Supplementary Material

btag737_Supplementary_Data
