# Supplementary material for: MAJEC: unified gene, isoform, and locus-level transposable element quantification from RNA-seq
Source: Bioinformatics. 2026 Oct 5;42(10):btag737. doi: 10.1093/bioinformatics/btag737 (PMC13676384; doi:10.1093/bioinformatics/btag737)
Supplement: Supplementary file 1 [file btag737_supplementary_data.docx]

**MAJEC: unified gene, isoform, and locus-level transposable element quantification from RNA-seq**

Tian-Yeh Lim^1^ and Ari J. Firestone^1#^

^1^Calico Life Sciences LLC, 1130 Veterans Blvd, South San Francisco, CA 94080

^#^To whom correspondence should be addressed. Phone: 1-650-726-7872. E-mail: firestone@calicolabs.com. Address: 1130 Veterans Blvd, South San Francisco, CA 94080.

**Figure S1. EM over-concentration at asymmetric isoform pairs.** **(A)** Violin plots of per-isoform absolute log2 fold error for each quantification method on the Sequins v2.4 dataset (Mix A and Mix B). The R2_7_1 outlier (circled, Mix A) represents MAJEC's worst-case failure. **(B)** Genomic structure of the R2_7 locus (chr18), showing two isoforms: R2_7_2 (7 exons, 1,728 bp) with 614,034 unique reads and 222,058 shared reads, and R2_7_1 (3 exons, 635 bp) with only 70 bp of unique territory and 2 unique reads. **(C)** Per-isoform count progression through MAJEC's prior adjustment stages. R2_7_2 receives large junction boosts from its exclusive junctions; R2_7_1 receives only a modest boost. The completeness penalty applies minor suppression to R2_7_1 (0.84), and the EM drives its final count to zero. **(D)** Final TPM estimates compared to ground truth (Mix A). MAJEC assigns nearly all signal to R2_7_2 (47,715 TPM vs ground truth 16,906) and zeroes R2_7_1 (3 TPM vs ground truth 16,906). Salmon distributes counts more evenly but still favors R2_7_2.

**Figure S2. LongBench isoform quantification benchmarking: per-cell-line and per-platform details.** **(A)** Forest plots of MAJEC's median error advantage over Salmon and RSEM (Left) for each cell line individually and pooled, with 95% bootstrap confidence intervals (consensus long-read ground truth). Superiority rates and Wilcoxon signed-rank significance (*** padj < 0.001 and at least one percentage point from parity) are shown. Right: Superiority rates pooled for all cell lines and stratified by long-read platform and by MAJEC junction penalty type. MAJEC's advantage is largest against dRNA ground truth and at subset-penalized transcripts; no significant advantage is observed for unpenalized transcripts. **(B)** Pairwise Pearson correlation heatmaps (log1p-transformed TPM) for each cell line across all quantification methods and long-read platforms. **(C)** MAJEC superiority rate stratified by ground truth expression level. MAJEC's advantage is largest at silent and low-expression transcripts and falls below 50% at mid-expression levels.

**Figure S3. Demonstration of MAJEC prior adjustment on synthetic data.** **(A)** IGV visualization of a synthetic locus containing three annotated transcripts of a synthetic gene: T1 (full-length, 6 exons), T2 (4 exons sharing E3 with T1 but using an alternative splice acceptor at E4'), and S1 (3 exons, a junction subset of T1 with a unique 5' extension). Coverage track (gray) and splice junction arcs (blue/green) are shown above transcript models. The red region (RT) marks S1's unique territory used for subset rescue. The green arc highlights T2's weakly supported junction versus T1's strongly supported E3→E4 junction at the same donor site. **(B)** MAJEC prior adjustment stages for junction completeness. Initial counts reflect featureCounts fractional assignment. Junction evidence boosts priors proportionally to splice junction support. The completeness penalty then downweights transcripts with poorly supported junctions relative to their gene context. **(C)** Subset penalty and territory rescue for S1. Because all S1's splice junctions are contained within T1 and show limited evidence of intendent S1 contribution, S1 receives a near-complete subset penalty. However, read coverage in S1's unique 5' territory (red region in A) partially rescues the prior, reflecting evidence that S1 may represent a real isoform despite its junction redundancy. Final EM counts: T1 = 1,572, T2 = 98, S1 = 18

**Figure S4. Parameter sensitivity analysis.** Each point shows the median log1p fold error across 8 cell lines against the consensus of long read data for a single parameter setting, with all other parameters held at their default values. The vertical gray line marks the baseline configuration. Parameters are grouped by feature: completeness penalty (overdispersion, min score), subset penalty (evidence threshold, penalty minimum, overdispersion), junction boost (weight, decay exponent), and other (paired rescue decay, terminal recovery rate). Default values: completeness overdispersion = 0.05, completeness min score = 0.0001, subset evidence threshold = 1.25, subset penalty minimum = 0.001, subset overdispersion = 0.1, junction weight = 3.0, junction decay exponent = 1.0, paired rescue decay = −1.5, terminal recovery rate = 2.0. Circles indicate swept values; triangles indicate conditions whose error falls beyond the plotted axis. Performance is broadly robust to parameter perturbation, with the exception of completeness overdispersion ≥ 0.1 and completeness min score ≥ 0.1, which substantially degrade accuracy by under-penalizing incomplete transcripts.

**Fig. S5. Intrinsic transcript features associated with MAJEC versus Salmon accuracy in LongBench.** For each transcript in each cell line, the tool with the lower absolute error (a per-transcript "win") was modeled against five standardized transcript features. The analysis is restricted to protein-coding and lncRNA transcripts, the biotypes reliably measured by long-read sequencing (n = 1,053,700 transcript-sample observations across 189,995 transcripts; overall MAJEC win rate 55.9%). **(A)** Odds ratios from a multivariable logistic regression for a MAJEC win, with z-scored predictors so each odds ratio is the change in odds of a MAJEC win per one standard-deviation (SD) increase in that feature. Odds ratios above 1 favor MAJEC (blue), below 1 favor Salmon (orange), the dashed line is OR = 1. Standard errors are cluster-robust by gene (32,403 clusters) to account for transcripts observed across multiple cell lines, and confidence intervals are narrow given the large n. **(B)** Proportional MAJEC win rate (ties counted as 0.5) by isoform count per gene, exon count, spliced-length quartile, and expression quintile, with bars colored by the winning tool and the dashed line marking 50% parity.

**Figure. S6. Benchmarking transcript-level quantification accuracy of MAJEC, Salmon, and RSEM using long-read ground truth.** Transcript quantification from short reads was compared against matched PacBio long-read data (quantified via IsoQuant) across three CD4 T cell activation timepoints (4hr, 16hr, and 48hr). **(A)** Cumulative distribution function (CDF) of absolute log-fold error for MAJEC (blue), Salmon (orange), and RSEM (green) relative to long-read counts. Transcripts are stratified by MAJEC’s algorithmically assigned prior penalty type (none, incomplete, subset, or both). **(B)** Paired comparisons of per-transcript error. The x-axis represents the median difference in absolute log-fold error (Δ error = MAJEC error – competitor error). Negative values indicate lower quantification error for MAJEC. *Left:* Median error differences pooled across all timepoints, stratified by penalty type. *Right:* Median error differences for all expressed transcripts, stratified by biological timepoint and pooled overall. Horizontal lines indicate 95% bootstrap confidence intervals for the median difference. Percentages denote MAJEC’s superiority rate (the percentage of transcripts where MAJEC achieved a lower absolute error than the competitor, with ties distributed equally). Statistical significance was assessed using one-sided paired Wilcoxon signed-rank tests on non-zero differences (*** p < 0.001 and at least one percentage point from parity). **(C)** MAJEC transcript-level superiority rate against Salmon (blue) and RSEM (red), stratified by long-read ground truth expression bins (Counts Per Million, CPM). The dashed grey line at 0.5 represents performance parity. Total transcript counts (*n*) per bin are annotated above the bars.

**Figure. S7. MAJEC versus Salmon accuracy and detection across sequencing depth in LongBench.** Short reads from three cell lines (SHP-77, NCI-H2228, NCI-H146) were downsampled to 7.5, 15, 30, and 55 M read pairs and re-quantified with each tool, holding the matched long-read consensus ground truth fixed. Isoforms were classified from the long-read data into three disjoint per-gene classes: dominant (the highest-abundance isoform), minor (non-dominant isoforms contributing ≥5% of the gene's long-read signal), and rare (non-dominant, <5%); n gives the per-line range of long-read-confirmed isoforms per class. Top row, median log-fold error; bottom row, detection sensitivity at a 0.1 TPM threshold (matching Fig 1E). Solid lines are medians across the three lines and faint points are individual lines. Lower error and higher sensitivity indicate better performance.

**Figure. S8. Comparison of MAJEC and TEtranscripts quantification and differential expression at the transposable element subfamily level.** **(A)** Correlation of raw read counts between MAJEC and TEtranscripts across the top eight most abundant transposable element (TE) classes. Subfamily-level counts from a representative sample were log10(x+1) transformed. The number of subfamilies (n) and Pearson correlation coefficients (r) are indicated for each class. Dashed red lines represent the line of identity. **(B)** Impact of the Expectation-Maximization (EM) algorithm on MAJEC’s subfamily-level quantification. Scatter plots display log10(x+1) transformed counts comparing pre-EM MAJEC (calculated as initial unique alignments plus fractional multimappers) versus TEtranscripts (left), final post-EM MAJEC versus TEtranscripts (middle), and the direct effect of the EM algorithm on MAJEC counts (right). Application of the EM framework maximizes concordance with TEtranscripts (post-EM r = 0.994). **(C)** Left: Correlation of DEseq2 log2 fold change values for the 61 subfamilies called discordantly significant between the MAJEC and TEtranscripts datasets. Right: Scatter plot of significance for the 61 discordant subfamilies showing -log10 of the DEseq2 adjusted p-values. The discordant calls are still highly correlated and are generally close to the significance thresholds.

**Figure. S9. RSEM comparison on locus-level TE quantification.** All values are counts over 4,532,102 TE loci in the shared gene+TE reference, pooled across all six samples (decitabine and DMSO, n = 3 each). **(A)** Number of TE loci receiving any signal, by locus length; a locus counts if the tool assigns it more than zero counts summed over the six samples. RSEM reports nothing below 150 nt. **(B)** Pearson correlation of log(1 + count) between each pair of tools, as a function of a minimum TE locus length cutoff. A locus contributes if either tool of the pair assigns it at least one count on average across the six samples. **(C)** Percentage of each tool's total TE signal originating from exon-overlapping loci, which make up 1.1% of annotated TE features. Solid bars use all loci; hatched bars restrict to ≥300 nt, the length range in which RSEM is able to participate**.**

**Figure. S10. Specificity on a TE-free (gene-only) simulation.** Paired-end reads (40M fragments, 150 bp) were simulated exclusively from GENCODE v44 protein-coding and lncRNA transcripts, with no TE sequence in the ground truth, so every read assigned to a TE locus is a false positive by construction. Telescope (US) is the released unstranded build; Telescope (stranded) is a development build. **(A)** False-TE assignment rate as a percentage of simulated fragments (log scale); fold values above the bars are relative to MAJEC. **(B)** TE subfamilies contributing the most false-positive fragments for MAJEC (left) and released Telescope (right) on a shared log scale, colored by family age (young L1, comprising L1HS and L1PA; ancient, all other families). Values are from one simulated library (sample_01).

**Figure. S11. Influence of gene overlap on locus-level transposable element quantification in joint versus TE-only models.** Transposable element (TE) loci were classified based on their genomic context relative to protein-coding genes: intergenic (blue), intronic (orange), or exon-overlapping (red). **(A)** Comparison of MAJEC run in its standard joint gene+TE mode versus a restricted TE-only mode (where gene annotations were excluded). *Top row:* Scatter plots of log10(x+1) transformed locus counts. The dashed grey line represents the line of identity. Pearson correlation coefficients (r) and the total number of loci with a count >= 1 in either model (n) are indicated. *Bottom row:* Histograms displaying the distribution of log2 fold changes (MAJEC joint / MAJEC TE-only) for expressed loci. The dashed grey line indicates a log2 fold change of zero (no difference), while the heavy dotted colored line marks the median difference (med). **(B)** Comparison of MAJEC (joint gene+TE model) against Telescope (an independent TE-only locus quantification tool). *Top row:* Scatter plots of log10(x+1) transformed locus counts across the three overlap classes. *Bottom row:* Histograms displaying the distribution of log2 fold changes (MAJEC / Telescope). As with the internal MAJEC comparison, exon-overlapping TEs show severe systemic disagreement (median log2FC = -2.58), highlighting the massive over-counting of genic reads by TE-only models when the joint feature space is ignored.

**Fig. S12. Genomic context of shared and tool-exclusive transposable element differential expression.** **(A)** Summary of differential expression (DE) calls within the shared feature space (39,050 TE loci that passed the minimum count threshold in both MAJEC and Telescope). The table displays the total number of loci per genomic class (intergenic, intronic, and exon-overlapping) alongside the number and percentage of significant DE calls (adjusted p-value < 0.05) made by each tool independently, as well as the concordant calls identified by both. **(B)** Composition of the exclusive feature space, defined as TE loci that passed the minimum expression filter in one tool but not the other. *Top row:* The proportion of all tool-exclusive loci assigned to each genomic overlap class for Telescope (left) and MAJEC (right). *Bottom row:* The proportion of significant DE calls (padj < 0.05) within those exclusive feature spaces. Total locus counts (*n*) and percentages are annotated within the bars. Consistent with the lack of a joint feature space, Telescope’s DE calls are heavily enriched for exon-overlapping and intronic elements, characteristic of host gene misattribution. In contrast, MAJEC suppresses these exonic counts, yielding an exclusive DE profile highly enriched for intergenic loci (58%) that represent unambiguous, autonomous TE transcription.

 **Fig. S13. Volcano plots of locus-level differential expression stratified by genomic context and shared versus exclusive feature spaces.** **(A)** Differential expression of transposable element (TE) loci quantified by Telescope. Plots display the log2 fold change (x-axis) against the -log10 adjusted p-value (y-axis). Columns separate loci based on their genomic context relative to protein-coding genes: intergenic (blue), intronic (orange), and exon-overlapping (red). The top row displays loci within the shared feature space (passing minimum count thresholds in both Telescope and MAJEC), while the bottom row displays Telescope-exclusive loci. The horizontal dashed grey line indicates the significance threshold (padj = 0.05). Total locus counts (n) and the number/percentage of significant DE calls (sig) are annotated above each plot. **(B)** Equivalent differential expression volcano plots for TE loci quantified by MAJEC, split into shared loci (top row) and MAJEC-exclusive loci (bottom row). Notably, Telescope generates a substantial number of highly significant, large-magnitude fold changes within its exclusive exon-overlapping feature space (A, bottom row). In contrast, the MAJEC-exclusive feature space exhibits a starkly flattened significance profile (B, bottom row), visually demonstrating that MAJEC’s joint EM framework successfully suppresses the spurious, high-significance fold changes driven by genic read misattribution.

**Fig. S14. Validation of Telescope false reactivation artifacts at the *IL11* locus following decitabine treatment.** **(A)** Stranded fractional read coverage across the *IL11* gene locus (chr19) in DMSO control (top) and decitabine treatment (middle) conditions. Plus-strand (blue) and minus-strand (red) coverage are plotted above gene and transposable element (TE) annotation tracks (bottom). Decitabine treatment induces massive minus-strand transcription that precisely matches the *IL11* exon-intron structure, with no independent transcriptional peaks isolated over the numerous embedded TE annotations. **(B)** Differential expression summary for all TE loci overlapping the *IL11* gene body. Rows highlighted in red represent loci that Telescope incorrectly reported as significantly upregulated (padj < 0.05) but which MAJEC correctly recognized as genic signal (either not tested due to insufficient counts or not significant). The log2 fold change and adjusted p-values are provided for each tool. Telescope systemically misattributes the drug-induced upregulation of the *IL11* gene to the embedded TE fragments.

**Table S1. Misattribution of TE reactivation signals to host genes by hierarchical quantification.** Top candidate loci demonstrating signal misattribution when using TEtranscripts count matrices. Candidates were selected based on identifying strong TE-driven upregulation (TE LFC > 1) that exceeded the host gene's signal magnitude. **MAJEC TE LFC** and **MAJEC Gene LFC** (along with corresponding p-adj values) reflect differential expression metrics calculated by DESeq2 using the probabilistically resolved count estimates from MAJEC's joint EM model. **TET Gene LFC** and **TET p-adj** reflect the corresponding DESeq2 results when using the count matrix generated by TEtranscripts. **LFC Inflation** is calculated as the difference between the TET Gene LFC and the MAJEC Gene LFC, representing the magnitude of false-positive gene signal generated by absorbed TE reads. Top misattributed loci are highlighted in red (Inflation > 1.0).

**Table S2. Detailed timing and computational scaling profile of the MAJEC pipeline.** Breakdown of wall time and scaling factors for each major phase of the MAJEC algorithm. Times reported reflect the joint processing of six RNA-seq samples (averaging **~53.5 million reads per sample**) on an Intel Xeon HPC cluster, totaling approximately 20 minutes. The underlying feature space is highly complex, comprising the full human reference transcriptome combined with RepeatMasker TE annotations for a total of **4,770,531 annotated features**. The execution time is dominated by BAM file I/O and alignment parsing via featureCounts (Phases 2a and 3), which scale linearly with input read depth. In contrast, the core mathematical modeling—including the joint momentum-accelerated EM algorithm (Phase 4a) and distinguishability metric calculations (Phase 4b)—is highly optimized. Despite tracking between 250,000 and 350,000 actively expressed features per sample, these joint modeling steps complete in under three minutes combined.
